# Supporting dataset and methods for body sizes and concentrations of chemical elements measured in elytra and abdomens of Stag Beetles *Lucanus cervus*

**DOI:** 10.1016/j.dib.2020.105935

**Published:** 2020-06-27

**Authors:** Grzegorz Orłowski, Lucyna Mróz, Marcin Kadej, Adrian Smolis, Dariusz Tarnawski, Jerzy Karg, Alessandro Campanaro, Marco Bardiani, Deborah J. Harvey, Marcos Méndez, Arno Thomaes, Al Vrezec, Krzysztof Ziomek, Andrzej L. Rudecki, Detlef Mader

**Affiliations:** a Institute of Agricultural and Forest Environment, Polish Academy of Sciences, Bukowska 19, PL-60-809 Poznań, Poland; b Department of Ecology, Biogeochemistry and Environmental Protection, Faculty of Biological Science, University of Wrocław, Kanonia 6/8, PL-50-328 Wrocław, Poland; c Department of Invertebrate Biology, Evolution and Conservation, Institute of Environmental Biology, Faculty of Biological Science, University of Wrocław, Przybyszewskiego 65, PL-51-148 Wrocław, Poland; d Faculty of Biological Sciences, Department of Nature Conservation, University of Zielona Góra, , Prof. Z. Szafrana 1, PL-65-516 Zielona Góra, Zielona Góra, Poland; e Consiglio per la ricerca in agricoltura e l'analisi dell'economia agraria – Centro di ricerca Difesa e Certificazione, Firenze, Italy; f Reparto Carabinieri Biodiversità di Verona, Centro Nazionale Carabinieri Biodiversità "Bosco Fontana", Mantova, Italy; g School of Biological Sciences, Royal Holloway University of London, Egham, UK; h Área de Biodiversidad y Conservacion, Universidad Rey Juan Carlos, Mostoles (Madrid), Spain; i Research Institute for Nature and Forest (INBO), Brussel, Belgium; j National Institute of Biology, Ljubljana, Slovenia; k Hebelstraße 12, D-69190 Walldorf, Germany

**Keywords:** Elemental composition, Trace elements, Internal metal concentrations, Exoskeleton, Chitin-bound metals

## Abstract

The dataset presented in this data paper supports “Breaking down insect stoichiometry into chitin-based and internal elemental traits: Patterns and correlates of continent-wide intraspecific variation in the largest European saproxylic beetle” (Orłowski et al. 2020). Here we present the supplementary data and description of methods on the following: (1) mass of elytra and abdomens across 28 local Stag Beetle *Lucanus cervus* populations in Europe. (2) Population origin and coverage of six major land-cover types, including transport infrastructure, measured in three radii (500 m, 1000 m and 5000 m) around the sampling sites of these populations. (3) The relationship between the mass and concentrations of elements measured in abdomens and elytra in 28 Stag Beetle populations and major land-cover types around the sampling sites.

Specifications table

**Table:** 

Subject	Ecotoxicology, Ecology, Biological Sciences
Specific subject area	Elemental composition, Saproxylic beetles
Type of data	Tables and Figures
How data was acquired	Through field work and laboratory work
Data format	Raw, filtered and analysed
Parameters for data collection	Investigation of abdomens (*n* = 124) and elytra (*n* = 271) of adult Stag Beetles collected at 28 localities situated in eight European countries from Central Spain to Western Russia.
Description of data collection	The measurement of concentrations of 12 chemical elements (Ca, Mg, K, Na, Mn, Fe, Zn, Cu, As, Cd, Pb and Ni), and relating these to insect size and local habitat conditions.
Data source location	Institution: Research Station of the Institute of the Agricultural and Forest Environment, Polish Academy of Sciences City/Town/Region: Turew, Wielkopolska Country: Poland
Data accessibility Related research article	All raw data are given along with the article as Appendix 1. G. Orłowski, L. Mróz, M. Kadej, A. Smolis, D. Tarnawski, J. Karg, A. Campanaro, M. Bardiani, D. J. Harvey, M. Méndez, A. Thomaes, A. Vrezec, K. Ziomek, A. L. Rudecki, D. Mader, Breaking down insect stoichiometry into chitin-based and internal elemental traits: Patterns and correlates of continent-wide intraspecific variation in the largest European saproxylic beetle. Environ. Poll. 262, 2020, 114,064.

## Value of the data

• This is the first published set of data on the variability of elemental traits (ETs), i.e. the concentrations of essential and non-essential elements, measured in the elytra and abdomens of Stag Beetles across the species’ distributional range in Europe.
• This dataset fills gaps in the environmental data relating to the accumulation of non-essential (toxic) elements in Stag Beetles.
• The data help to understand the differentiation between the elemental pool of metals accumulated in the exoskeleton and internal organs, including the residual body fat of holometabolous insects.
• The data can be used to determine the extent and impact of the coverage of major land-cover types, including transport infrastructure, on ETs of the exoskeletons and internal organs of beetles.
• The data may be useful for explaining the sources of variation in internal and exoskeletal ETs of beetles and other insects, and provide a basis for further ecotoxicology work.

## 1 Data description

**Fig. 1:**
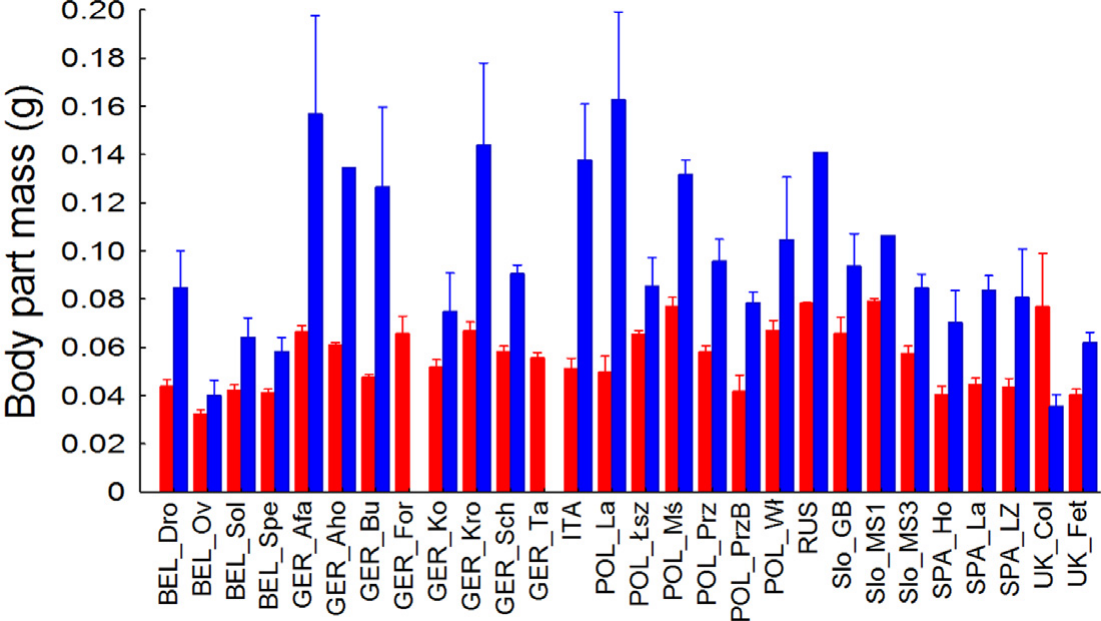
Comparison (average ± SE) of mass of elytra (red columns; *n* = 271) and abdomens (blue columns; *n* = 124) across 28 local Stag Beetle *Lucanus cervus* populations in Europe. Notes: (1) The masses of elytra (F_27,__243_ = 2.94, *P* < 0.001) and abdomens (F_25,__98_ = 4.18, *P* < 0.001) both vary significantly among these populations. Similarly, the masses of both these body parts differ significantly per individual population across all these sites (body part × population interaction term: F_27,__367_ = 2.41, *P* < 0.001). (2) The masses of elytra and abdomens (for each *n* = 26) were both positively correlated with the longitudes (for georeferences, see Table 1) of the sampling sites (Pearson *r* = 0.583 and 0.491, *P* = 0.002 and 0.011; respectively), but not with their latitudes (*r* = 0.228 and 0.056, *P* = 0.262 and 0.787; respectively). However, because of the small sample size and no sexing of the beetles, the results of this analysis should be treated with caution: they are presented only for the purposes of this paper, and may not represent the actual variation in body size of *L. cervus* across Europe.

**Photo 1:**
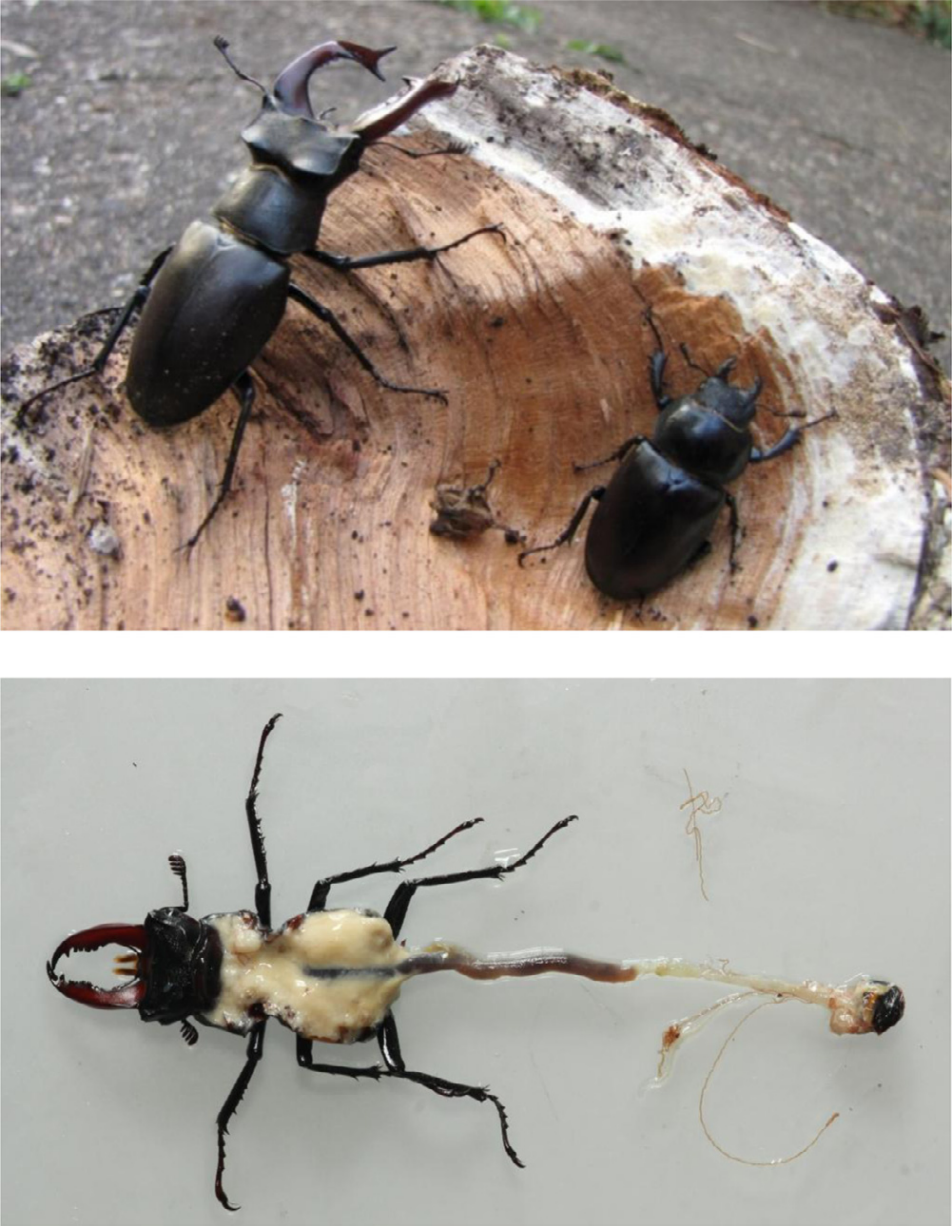
The European Stag Beetle *Lucanus cervus*. **Top:** Sexual dimorphism; left to right – male imago, body size 46 mm, and female imago, body size 37 mm. **Bottom:** dissection of a teneral imago, body size 36 mm, showing the fat body (white), and part of the alimentary canal. The midgut, 45 mm, has food remnants from the larval stage, the hind gut, ∼24 mm, is clear. The hind gut is relatively short, a characteristic of sap feeders. In the adult, food intake, if any, is restricted to sap runs; but it can be tempted to sugary liquids. Photos© Maria Fremlin.

The data presented here (Photo 1; Fig. 1; Table 1, Table 2, Table 3, Table 4; Appendix A) constitute the basis for the article by Orłowski et al. [1]. The dataset provides detailed information on: (1) the masses of elytra and abdomens across 28 local Stag Beetle *Lucanus cervus* (Photo 1) populations in Europe (Fig. 1); (2) the population origin and coverage of six major land-cover types, including transport infrastructure, measured in three radii (500 m, 1000 m and 5000 m) around the sampling sites of these populations (Tables 1 and 2); (3) the relationship between the mass and concentrations of elements measured in abdomens and elytra in 28 Stag Beetle populations and major land-cover types around the sampling sites (Tables 3 and 4). The raw data with the concentrations of 12 chemical elements measured in elytra and abdomens of Stag Beetles from 28 European populations are presented in Appendix A.

**Table 1:** Population origin and coverage of six major land-cover types, including transport infrastructure, measured in three radii (500 m / 1000 m / 5000 m) around the sampling sites of 28 Stag Beetle *Lucanus cervus* populations; for sample size, see Table 2. Note: the total areas in consecutive radii are 500 *m* = 78.5 ha, 1000 *m* = 314 ha and 5000 *m* = 7 850 ha. ^1^Includes artificial non-agricultural areas such as lawns, and sport and leisure areas.

Country	Site = Village or town name/Conventional description	Population (acronym)	Latitude, longitude	Urban/ built-up areas (ha)	Wood/park/forest (ha)	Agriculturalland/ cultivation (ha)	Grassland/ open habitats with low vegetation (ha)^1^	Roads/railways (ha)	Water/ lakes/ river (ha)
Belgium	Speelberg (suburban)	BEL_Spe	50.761152, 4.530245	27.9/29.2/3838.3	1.7/50.2/1120.8	47.1/197.9/2539.6	0.0/23.9/211.4	1.8/11.0/67.6	0.0/1.9/72.4
	Overijse (Putterstr) (suburban)	BEL_Ov	50.777305, 4.545712	27.3/112.8/2809.4	19.7/61.1/1777.6	26.0/124.5/3056.3	0.0/0.0/135.3	1.8/4.6/39.7	3.7/11.0/31.7
	Solheide (suburban)	BEL_Sol	50.774054, 4.535564	64.9/ 167.7/2924.9	3.2/57.8/1832.2	1.9/57.9/2862.3	4.9/19.3/142.3	1.9/6.6/56.0	1.8/4.7/32.4
	Drogenberg (suburban)	BEL_Dro	50.771654, 4.528396	40.9/151.1/2945.3	25.7/85.8/1998.7	0.0/44.2/2622.2	7.4/18.0/158.4	1.5/7.3/62.9	2.9/7.6/62.6
Germany	Bullay (suburban)	GER_Bu	50.053653, 7.133485	31.8/92.0/2447.8	3.3/89.2/2230.5	3.4/60.4/1509.5	7.9/30.0/690.7	4.9/3.6/29.5	27.2/38.9/971.5
	Alf (Holzplatz) (suburban)	GER_Aho	50.050175, 7.104856	9.6/34.3/1184.8	61.8/229.5/5737.3	4.9/33.2/829.0	0.0/0.0/0.0	2.1/16.4/82.0	0.0/0.7/16.9
	Alf (Fabrik) (suburban)	GER_Afa	50.058964, 7.106158	1.6/33.6/840.8	68.8/195.1/4877.6	0.0/61.6/1538.8	5.8/22.8/571.0	1.3/0.0/0.0	1.1/0.9/21.8
	Samtgemeinde Schüttorf (suburban)	GER_Sch	52.327063, 7.257903	3.1/39.1/994.5	36.6/91.6/2288.9	31.2/120.5/3012.1	5.3/43.7/1093.7	2.1/11.1/262.5	0.1/7.9/198.3
	Koblenz (suburban)	GER_Ko	50.348776, 7.580741	18.2/199.7/4992.7	50.4/82.8/2068.8	0.0/0.0/0.0	1.9/6.4/159.3	8.0/25.0/624.4	0.0/0.2/4.8
	Tairnbach (suburban)	GER_Ta	49.255642, 8.750627	9.7/38.6/964.2	51.2/138.1/3452.2	13.5/108.0/2699.9	2.7/18.8/469.0	1.4/1.9/48.7	0.1/8.6/216.0
	Kronau (suburban)	GER_Kro	49.215231, 8.620142	52.0/125.3/3132.6	1.6/37.0/926.1	16.0/129.5/2714.8	8.0/0.0/0.0	0.9/5.6/140.2	0.0/16.6/414.5
	Forst (suburban)	GER_For	49.147766, 8.575710	33.2/84.2/2120.8	25.3/105.7/2641.6	5.7/83.3/2082.5	6.9/25.4/635.5	3.1/9.6/225.9	4.3/5.7/143.6
Italy	Marmirolo (forest)	ITA	45.200637, 10.745660	0.7/1.5/1078.9	59.6/214.8/238.8	0.0/82.1/6071.8	17.8/13.7/90.7	0.0/0.0/0.0	0.4/1.9/391.8
Poland	Przemków, Beech Forest reserve	POL_PrzB	51.508000, 15.693300	0.0/0.0/175.4	78.5/289.9/3747.0	0.0/0.0/3927.6	0.0/24.1/27.1/	0.0/0.0/0.0	0.0/0.0/0.0
	Przemków (forest)	POL_Prz	51.516150, 15.861000	0.0/0.0/56.0	78.5/314.0/6845.0	0.0/0.0/949.1	0.0/0.0/0.0	0.0/0.0/0.0	0.0/0.0/0.0
	Mścigniew (forest)	POL_Mś	51.914931, 16.326594	0.0/0.0/321.2	77.6/297.7/5015.7	0.0/13.9/2092.2	0.0/0.0/0.0	0.9/2.4/11.9	0.0/0.0/409.0
	Włoszakowice (forest)	POL_Wł	51.897786, 16.336065	0.0/0.0/201.0	78.5/314.7/5473.4	0.0/0.0/2123.0	0.0/0.0/0.0	0.0/0.0/0.0	0.0/0.0/52.5
	Ławszowa Jeziory (forest)	POL_Łsz	51.397417, 15.455117	0.0/0.0/26.1	78.5/314.0/880.1	0.0/0.0/499.1	0.0/0.0/444.7	0.0/0.0/0.0	0.0/0.0/0.0
	Lasowice Milicz (forest)	POL_La	51.435584, 17.254254	0.0/0.0/79.5	77.6/312.0/6836.7	0.0/0.0/923.9	0.0/0.0/0.0	0.9/2.0/9.9	0.0/0.0/0.0
Russia	Kursk (forest)	RUS	51.146944, 36.430000	0.0/0.0/148.5	14.3/160.4/852.1	57.7/109.0/4876.9	6.5/44.7/1972.5	0.0/0.0/0.0	0.0/0.0/0.0
Slovenia	Gornja Bistrica (forest)	Slo_GB	46.547811, 16.260903	0.7/27.5/686.3	69.4/202.1/5069.9	3.0/59.2/1479.9	0.0/9.2/230.4	0.0/1.4/34.1	5.4/14.7/367.8
	Murska šuma 1 (forest)	Slo_MS1	46.493156, 16.52374	0.0/0.0/0.0	62.0/283.2/7080.4	0.0/0.0/0.0	8.7/10.6/265.2	0.0/0.0/0.0	7.8/20.2/504.4
	Murska šuma 3 (forest)	Slo_MS3	46.504614, 16.510087	0.0/2.9/72.8	41.2/159.9/3997.3	37.3/146.8/3671.0	0.0/0.0/0.0	0.0/0.0/0.0	0.0/4.4/108.9
Spain	La Laguna, Asturias (forest)	SPA_La	43.514092, −5.958645	9.3/15.6/389.9	32.6/102.5/2562.3	33.1/141.1/3527.1	2.0/50.6/1265.8	1.5/4.2/105.0	0.0/0.0/0.0
	Lozoya river Spain (forest)	SPA_LZ	40.900844, −3.457267	0.0/0.0/0.0	15.3/34.1/65.8	0.0/0.0/0.0	60.0/271.1/6778.2	1.0/3.6/90.1	2.3/5.1/981.7
	Horcajo de la Sierra (forest)	SPA_Ho	41.068931, −3.579413	1.9/8.2/205.9	8.8/82.7/1011.7	24.7/0.0/1054.8	41.8/219.6/5490.0	1.4/3.5/87.7	0.0/0.0/0.0
United Kingdom	Colchester (urban)	UK_Col	51.883472, 0.883527	58.8/235.3/5881.5	3.7/19.0/476.1	0.0/0.0/0.0	11.4/51.0/1275.1	4.6/8.7/217.3	0.0/0.0/0.0
	Fetcham (urban)	UK_Fet	51.297159, −0.351009	37.5/162.1/4052.4	14.5/33.2/860.0	9.4/58.0/1451.2	5.3/46.9/1172.2	7.9/10.2/255.0	4.0/3.6/88.8

**Table 2:** Descriptive statistics (median; range) of concentrations of 12 chemical elements measured in elytra (white rows) and abdomens (blue rows) of Stag Beetles *Lucanus cervus* from 28 European populations of the species; see Table 1 for population acronyms; the last two rows contain data pooled across all the populations.

Population	Number of samples	Ca			Mg			K			Na			Mn							
		Med	Min	Max	Med	Min	Max	Med	Min	Max	Med	Min	Max	Med	Min	Max					
BEL_Dro	8	378.1	185.4	516.7	593.1	455.1	889.0	2068.1	1133.2	2293.6	273.7	215.9	442.9	6.6	5.0	7.7					
BEL_Dro	3	812.6	465.6	984.7	5342.7	3268.8	5537.5	7085.7	6123.4	9135.3	713.7	707.0	1043.4	14.0	9.2	17.7					
BEL_Ov	4	452.8	317.3	1765.0	437.6	311.2	627.1	1765.6	784.2	2847.4	561.0	198.4	1288.7	5.9	2.9	23.9					
BEL_Ov	2	930.8	567.2	1294.5	2121.0	1313.9	2928.1	5839.1	1816.1	9862.1	1065.0	353.1	1776.9	20.3	19.3	21.3					
BEL_Sol	12	510.1	290.2	2672.5	554.5	348.7	2036.5	1919.9	661.1	2683.9	247.7	119.8	447.2	17.3	6.4	96.2					
BEL_Sol	6	1324.2	664.9	2509.7	3220.7	809.6	10,347.7	6390.8	945.5	7658.4	627.4	294.4	687.9	22.6	12.1	31.0					
BEL_Spe	33	476.5	257.8	3629.5	570.1	333.8	1307.9	2466.7	496.6	4925.9	398.0	142.6	630.0	13.6	6.2	44.6					
BEL_Spe	19	669.9	445.2	2940.5	2817.1	1514.1	9611.3	6458.7	3247.0	9743.8	821.1	457.9	1283.0	18.9	11.9	62.4					
GER_Afa	4	302.3	200.4	481.9	692.2	641.2	777.4	3917.8	2070.6	5828.2	753.1	646.1	795.4	34.3	26.6	48.8					
GER_Afa	2	425.7	364.2	487.2	2951.6	1920.0	3983.3	10,678.2	10,293.7	11,062.7	1993.7	1744.2	2243.2	64.7	54.9	74.5					
GER_Aho	4	357.9	351.9	367.8	542.4	417.1	661.7	2754.2	2704.0	2997.1	498.0	423.7	528.7	24.2	19.7	27.9					
GER_Aho	1	483.3	483.3	483.3	4175.6	4175.6	4175.6	6214.7	6214.7	6214.7	986.8	986.8	986.8	35.4	35.4	35.4					
GER_Bu	6	393.9	286.6	420.9	732.8	471.2	1050.9	5182.3	4326.2	7253.5	899.5	755.1	1022.2	21.0	16.9	24.3					
GER_Bu	3	1100.4	1006.0	1662.5	8016.3	5888.1	10,211.5	11,909.8	11,175.9	15,927.7	2520.9	1949.1	2572.8	64.2	49.7	85.6					
GER_For	6	417.3	335.6	588.8	432.5	292.9	637.0	3252.2	2595.0	3777.8	805.8	594.9	1103.9	11.5	9.9	20.5					
GER_Ko	11	359.2	295.1	482.5	704.7	392.3	926.3	2317.8	1075.7	3836.8	750.6	486.4	1066.3	20.6	9.5	25.2					
GER_Ko	4	583.4	387.1	720.8	3207.3	2106.6	8601.8	10,087.6	4454.2	11,908.2	1538.4	1009.0	1798.1	22.2	18.3	32.5					
GER_Kro	9	474.9	376.2	1573.6	693.0	368.6	1012.9	3218.7	2032.3	4358.5	875.6	748.2	1930.6	12.9	10.9	23.4					
GER_Kro	2	704.6	676.1	733.1	2158.9	1551.9	2765.9	8982.1	8167.2	9797.1	2081.2	2003.0	2159.5	16.6	15.9	17.3					
GER_Sch	6	360.1	338.0	407.7	654.9	576.5	819.9	2847.3	2539.6	3877.3	872.0	622.8	1300.9	15.0	9.8	24.8					
GER_Sch	2	675.2	639.6	710.8	5921.5	2612.1	9230.8	10,510.0	6717.5	14,302.5	1778.1	1402.0	2154.2	37.7	24.7	50.7					
GER_Ta	18	378.7	265.1	1092.2	578.2	464.8	1083.2	2559.8	808.8	5686.5	886.9	335.2	1237.3	12.1	6.6	19.8					
ITA	11	598.5	311.8	3755.3	819.3	504.7	1689.0	1294.8	977.8	3348.4	628.1	279.0	1204.0	21.1	16.4	31.3					
ITA	7	837.8	521.8	4634.5	4482.7	2495.9	6416.5	6016.0	5425.5	14,147.8	826.2	600.9	2612.2	17.6	10.4	29.2					
POL_La	4	341.7	242.3	1765.1	664.4	582.5	1074.0	2246.3	1570.2	4884.9	353.0	104.2	1237.5	38.8	19.0	84.3					
POL_La	3	361.0	266.3	439.2	5903.1	1507.2	13,474.6	11,696.2	6895.8	12,331.8	959.4	876.2	2003.1	29.6	13.3	31.9					
POL_Łsz	12	285.0	251.6	383.3	563.1	447.8	641.6	3966.5	3260.4	6522.9	724.8	540.0	1158.1	16.0	6.9	20.2					
POL_Łsz	6	504.2	231.3	1200.0	2119.4	974.7	4091.6	11,715.1	6245.5	29,765.8	1844.1	1069.0	4018.0	63.4	23.9	109.0					
POL_Mś	6	286.7	246.7	848.1	480.8	421.9	625.1	3721.6	3252.4	4969.1	959.7	725.7	1088.3	19.9	12.5	27.0					
POL_Mś	3	469.4	456.4	662.5	1913.4	1379.7	1970.2	12,022.9	11,369.6	17,210.4	1954.6	`	2022.9	35.7	28.1	53.0					
POL_Prz	30	335.4	219.3	2444.6	524.1	120.2	764.9	3249.1	529.3	5485.5	822.5	82.1	1725.2	21.7	11.1	321.0					
POL_Prz	15	512.5	216.4	1300.3	2551.8	956.5	5599.7	15,762.7	1126.3	20,761.3	2081.6	81.2	2677.8	78.7	27.8	623.2					
POL_PrzB	4	483.6	337.6	572.8	898.0	559.2	1152.0	3157.5	2793.8	8913.1	626.8	564.3	694.3	30.0	28.4	35.3					
POL_PrzB	2	387.1	297.3	476.9	2315.3	1592.8	3037.9	11,812.4	8778.8	14,846.0	1308.0	1071.3	1544.7	39.6	34.9	44.2					
POL_Wł	10	382.1	229.5	513.2	599.2	451.2	872.5	3445.8	2511.6	4616.5	1314.3	701.8	1744.7	29.3	19.4	41.5					
POL_Wł	6	489.3	327.5	755.4	2472.3	780.6	9191.9	10,039.3	3625.7	15,077.2	1601.5	791.3	2410.5	44.2	26.4	56.9					
RUS	2	290.3	262.0	318.6	544.2	505.3	583.2	2807.9	2650.6	2965.1	1891.9	1571.4	2212.5	8.4	7.2	9.6					
RUS	1	922.7	922.7	922.7	1534.5	1534.5	1534.5	9521.6	9521.6	9521.6	2356.4	2356.4	2356.4	19.1	19.1	19.1					
Slo_GB	4	250.6	166.6	355.8	158.8	143.7	163.7	534.8	404.5	846.2	126.1	119.3	137.0	9.1	7.8	11.1					
Slo_GB	2	502.5	437.1	567.9	521.6	391.3	651.9	903.8	789.5	1018.2	227.4	196.8	258.0	14.5	14.3	14.7					
Slo_MS1	2	231.7	207.7	255.7	229.7	190.2	269.3	576.5	464.0	688.9	151.6	119.9	183.3	11.4	11.3	11.5					
Slo_MS1	1	290.0	290.0	290.0	414.4	414.4	414.4	688.0	688.0	688.0	188.4	188.4	188.4	20.4	20.4	20.4					
Slo_MS3	10	313.1	248.1	434.1	208.6	156.5	366.8	1141.4	898.2	1608.5	211.6	165.4	351.5	12.9	9.6	21.9					
Slo_MS3	5	579.3	519.5	623.7	577.2	424.1	823.2	2341.1	2201.5	2880.4	439.9	358.3	452.2	18.9	15.1	27.0					
SPA_Ho	7	296.6	267.1	642.4	926.8	371.3	1007.5	2250.6	1993.2	4658.9	969.2	371.6	1044.7	17.1	13.8	23.3					
SPA_Ho	4	506.4	384.7	606.2	2914.3	1813.3	4954.0	8765.3	5153.2	12,765.2	1244.9	1010.0	1543.9	29.6	23.0	42.6					
SPA_La	17	415.3	271.5	534.4	810.0	656.8	1040.5	1559.0	854.5	2737.9	717.8	356.6	1239.5	15.5	7.9	33.0					
SPA_La	8	445.4	368.7	686.5	3092.1	1755.2	7268.8	5793.4	4691.8	8711.0	1111.1	834.7	2666.1	19.1	9.0	22.5					
SPA_LZ	5	278.2	222.3	359.0	1091.9	609.0	1177.8	2511.0	1634.7	2745.5	731.2	678.5	1660.9	20.6	18.6	23.3					
SPA_LZ	3	399.0	391.3	556.4	5496.8	1258.7	6007.1	9316.9	5145.2	9947.4	1084.3	1065.1	1707.9	32.0	21.3	42.0					
UK_Col	14	329.1	27.6	711.4	683.7	24.3	1080.0	2536.4	207.2	5760.6	884.0	37.5	1410.8	15.5	0.8	22.8					
UK_Col	7	1315.0	781.5	2684.7	4165.9	720.0	16,348.5	7025.6	2231.3	14,496.1	902.2	556.0	2736.4	44.1	20.3	70.9					
UK_Fet	12	329.4	258.9	659.0	500.1	435.2	576.8	3755.4	2300.4	7172.3	979.8	611.5	2027.7	13.3	9.0	21.2					
UK_Fet	7	1160.6	891.3	1680.7	3957.3	2869.0	8756.4	15,183.8	11,191.2	20,611.8	2867.6	2090.9	5717.1	30.1	23.0	51.2					
Totals	271	367.5	27.6	3755.3	591.2	24.3	2036.5	2683.9	207.2	8913.1	707.5	37.5	2212.5	17.2	0.8	321.0					
Totals	124	610.3	216.4	4634.5	3016.2	391.3	16,348.5	8349.1	688.0	29,765.8	1206.2	81.2	5717.1	26.9	9.0	623.2					
Population	Fe			Zn			Cu			As			Cd			Pb			Ni		
	Med	Min	Max	Med	Min	Max	Med	Min	Max	Med	Min	Max	Med	Min	Max	Med	Min	Max	Med	Min	Max

BEL_Dro	29.6	17.3	52.7	11.1	0.0	38.6	4.0	3.2	4.5	0.6	0.24	2.2	0.008	0.004	0.022	0.385	0.23	1.72	0.17	0.044	0.31
BEL_Dro	156.7	153.5	323.1	170.6	156.3	210.9	18.6	12.5	27.4	2.7	2.68	16.9	0.019	0.018	0.094	0.572	0.55	1.17	0.42	0.217	0.64
BEL_Ov	125.6	31.9	966.7	51.5	5.9	54.5	7.7	7.4	9.5	3.8	0.94	4.3	0.035	0.024	0.082	1.152	0.80	3.58	5.00	1.999	9.20
BEL_Ov	544.7	221.1	868.2	322.2	96.6	547.8	36.0	13.2	58.8	34.2	17.25	51.1	0.056	0.054	0.059	1.639	1.48	1.80	2.20	1.782	2.62
BEL_Sol	135.5	7.1	2220.8	14.1	3.0	43.5	6.7	3.5	17.1	1.4	0.39	5.7	0.020	0.006	0.171	1.114	0.43	9.80	1.08	0.358	3.49
BEL_Sol	282.3	146.2	943.6	239.9	97.2	348.9	35.9	21.3	95.7	19.4	2.26	88.0	0.052	0.040	0.138	1.719	0.84	32.14	0.58	0.339	0.90
BEL_Spe	32.2	11.7	1141.9	14.5	0.0	73.4	5.3	3.8	15.6	1.4	0.21	8.7	0.026	0.008	0.094	0.714	0.23	7.86	0.37	0.075	6.29
BEL_Spe	103.2	45.9	1243.1	218.5	110.7	526.0	28.6	10.5	156.1	22.5	1.55	186.9	0.066	0.030	0.370	0.991	0.55	3.95	0.42	0.150	7.60
GER_Afa	317.3	69.6	1075.1	29.9	8.8	54.3	6.7	5.4	6.9	2.2	1.52	2.9	0.015	0.010	0.019	0.607	0.34	1.45	1.21	0.957	1.82
GER_Afa	812.4	783.3	841.5	170.9	123.9	217.9	58.9	21.7	96.1	9.6	1.09	18.0	0.026	0.021	0.031	1.183	0.93	1.43	1.41	1.405	1.41
GER_Aho	44.0	28.9	64.2	6.1	5.5	7.0	3.9	3.7	4.8	0.8	0.61	0.9	0.012	0.006	0.014	0.299	0.14	0.43	0.33	0.172	0.46
GER_Aho	297.1	297.1	297.1	119.0	119.0	119.0	20.2	20.2	20.2	1.3	1.27	1.3	0.017	0.017	0.017	0.223	0.22	0.22	0.31	0.307	0.31
GER_Bu	96.7	73.4	303.1	40.2	20.8	54.0	10.5	7.2	16.8	4.5	2.28	8.5	0.013	0.013	0.018	0.718	0.44	3.79	1.03	0.572	1.48
GER_Bu	668.1	167.0	1125.7	193.1	119.7	302.1	136.4	106.5	219.1	18.5	0.09	38.5	0.017	0.007	0.048	1.744	0.39	2.10	0.80	0.172	1.24
GER_For	28.3	14.7	72.0	30.2	13.3	53.4	3.3	2.9	3.6	0.9	0.45	1.3	0.005	0.003	0.007	0.541	0.27	0.94	0.31	0.154	1.30
GER_Ko	26.9	13.6	102.3	8.3	0.8	46.7	4.7	3.0	8.8	1.4	0.23	11.6	0.013	0.004	0.027	0.269	0.15	1.19	0.26	0.169	0.98
GER_Ko	123.3	76.9	215.6	247.3	91.7	407.2	70.4	53.4	125.3	21.7	7.58	59.4	0.040	0.016	0.065	0.334	0.18	1.05	0.28	0.056	0.41
GER_Kro	35.1	23.7	121.5	8.5	4.0	17.5	4.7	3.5	5.7	0.7	0.51	2.4	0.006	0.003	0.011	0.534	0.32	1.23	0.50	0.370	0.86
GER_Kro	122.5	67.3	177.8	153.1	145.2	161.0	49.4	43.2	55.6	14.2	1.60	26.7	0.017	0.006	0.027	0.237	0.20	0.27	0.23	0.224	0.24
GER_Sch	24.6	13.2	59.8	8.8	0.2	22.0	5.9	3.1	7.6	4.5	0.35	5.6	0.017	0.012	0.020	0.387	0.15	0.58	0.54	0.389	1.05
GER_Sch	270.2	221.0	319.5	247.5	223.4	271.6	114.9	51.8	178.0	97.6	57.44	137.7	0.098	0.059	0.137	0.961	0.64	1.29	0.64	0.510	0.77
GER_Ta	30.1	11.8	83.2	5.0	0.000	32.2	3.6	2.8	5.9	0.8	0.22	3.1	0.007	0.004	0.019	0.489	0.26	1.08	0.41	0.101	1.45
ITA	24.1	13.6	289.1	13.7	6.6	60.2	4.3	2.9	4.7	1.4	0.40	2.5	0.020	0.013	0.031	0.438	0.24	32.60	0.27	0.134	0.43
ITA	140.4	68.6	469.7	148.7	88.3	283.5	36.9	25.6	134.7	14.7	3.34	39.0	0.029	0.013	0.083	0.198	0.02	1.60	0.08	0.054	0.54
POL_La	45.7	30.0	76.2	27.9	12.2	105.3	4.0	3.8	17.1	1.2	0.84	2.5	0.016	0.007	0.064	0.784	0.42	1.41	1.20	0.933	2.00
POL_La	217.5	110.5	366.5	143.4	101.8	257.3	30.3	21.7	98.5	17.6	1.57	28.4	0.020	0.019	0.113	0.557	0.27	1.05	0.30	0.239	6.04
POL_Łsz	25.1	16.0	45.1	14.3	7.7	44.1	4.8	3.1	7.5	1.8	0.64	7.1	0.009	0.003	0.018	0.424	0.24	0.70	0.38	0.065	1.02
POL_Łsz	151.0	98.0	193.2	235.4	166.5	300.9	59.1	41.8	188.7	19.3	4.35	66.9	0.038	0.024	0.083	0.668	0.44	1.26	0.38	0.274	4.92
POL_Mś	35.1	23.6	280.3	21.7	17.0	33.6	4.6	4.3	5.6	1.1	0.25	4.2	0.036	0.021	0.098	0.867	0.68	30.77	0.38	0.264	0.64
POL_Mś	130.4	102.5	203.3	212.5	211.9	232.8	155.4	123.6	181.2	22.8	4.61	79.6	0.045	0.021	0.052	0.622	0.59	0.70	0.20	0.152	0.22
POL_Prz	15.8	8.9	134.0	16.7	5.4	54.1	4.6	3.4	129.6	1.2	0.38	12.7	0.020	0.008	0.118	0.353	0.14	4.23	0.17	0.020	1.81
POL_Prz	85.4	45.8	431.0	232.8	54.6	457.8	73.5	22.9	98.5	40.2	0.00	78.0	0.046	0.025	0.184	0.689	0.24	13.02	0.11	0.031	1.74
POL_PrzB	197.8	75.7	310.0	26.8	19.4	45.2	8.7	6.1	19.3	1.5	0.002	3.0	0.078	0.000	0.212	2.445	2.11	3.16	1.18	1.018	1.45
POL_PrzB	397.5	382.3	412.7	259.2	212.2	306.1	82.9	61.3	104.4	32.6	29.16	36.0	0.132	0.115	0.150	2.237	2.06	2.42	1.18	0.677	1.68
POL_Wł	26.3	18.3	50.9	6.5	4.9	10.3	4.6	3.6	7.6	1.2	0.77	2.6	0.009	0.005	0.047	0.437	0.32	0.75	0.30	0.206	0.74
POL_Wł	124.4	105.4	216.0	217.9	72.8	273.0	59.6	31.9	211.2	19.3	11.61	144.7	0.020	0.003	0.051	0.962	0.48	1.35	0.21	0.015	3.05
RUS	24.0	19.1	28.9	12.0	12.0	12.1	4.2	4.2	4.3	0.2	0.19	0.2	0.005	0.004	0.006	0.263	0.22	0.30	0.19	0.158	0.22
RUS	124.2	124.2	124.2	180.8	180.8	180.8	52.4	52.4	52.4	2.5	2.48	2.5	0.023	0.023	0.023	0.310	0.31	0.31	0.22	0.217	0.22
Slo_GB	25.5	20.8	26.2	15.7	7.8	29.1	6.3	5.3	8.7	2.8	0.42	6.1	0.006	0.003	0.008	0.260	0.15	0.34	0.35	0.302	1.15
Slo_GB	132.9	113.0	152.7	65.4	27.6	103.2	42.8	37.8	47.8	12.2	1.29	23.1	0.013	0.010	0.016	0.252	0.17	0.33	0.56	0.384	0.74
Slo_MS1	39.4	36.3	42.5	28.0	25.8	30.2	5.2	5.1	5.3	4.7	4.43	4.9	0.009	0.009	0.009	0.210	0.20	0.22	0.66	0.526	0.80
Slo_MS1	817.7	817.7	817.7	55.7	55.7	55.7	40.9	40.9	40.9	12.6	12.56	12.6	0.039	0.039	0.039	0.550	0.55	0.55	0.30	0.297	0.30
Slo_MS3	33.0	23.8	47.1	20.3	10.4	36.1	6.7	5.4	12.0	1.9	1.02	5.8	0.015	0.011	0.023	0.375	0.19	1.80	0.50	0.169	4.40
Slo_MS3	130.4	120.4	286.4	57.0	43.9	71.5	43.2	32.3	59.1	5.9	1.99	18.9	0.080	0.057	0.105	0.311	0.22	0.34	0.42	0.158	1.13
SPA_Ho	145.3	90.8	267.5	19.5	16.1	30.5	5.0	4.0	6.9	2.7	0.35	37.3	0.017	0.005	0.070	0.674	0.44	0.80	0.37	0.169	0.85
SPA_Ho	471.0	131.8	935.5	228.8	159.3	315.0	66.7	46.3	75.2	15.9	0.97	70.0	0.055	0.020	0.093	0.680	0.19	0.76	0.51	0.269	0.71
SPA_La	50.0	24.7	322.5	13.7	3.5	156.6	4.7	2.9	9.1	0.8	0.04	5.1	0.041	0.021	0.202	0.838	0.36	9.81	0.18	0.020	1.58
SPA_La	166.1	42.7	819.7	142.3	86.6	345.4	31.2	8.6	80.1	12.4	0.52	83.5	0.090	0.059	0.586	0.641	0.36	1.21	0.19	0.107	1.11
SPA_LZ	92.5	78.9	108.0	23.9	15.8	29.3	4.7	4.4	5.2	7.8	1.45	20.5	0.025	0.014	0.065	0.427	0.31	0.62	0.47	0.066	5.96
SPA_LZ	223.9	161.1	372.2	156.5	124.0	195.7	54.4	48.8	76.1	119.5	26.77	254.9	0.042	0.016	0.061	0.346	0.32	0.72	0.16	0.143	0.37
UK_Col	42.5	7.8	160.4	17.5	0.6	75.4	5.7	1.2	15.0	2.7	0.56	45.0	0.041	0.004	0.093	1.183	0.23	4.99	0.07	0.003	0.63
UK_Col	351.5	161.8	2522.3	422.0	116.4	658.6	74.0	48.6	105.8	56.6	1.88	232.0	0.107	0.048	0.161	10.8	3.92	32.67	1.10	0.220	2.24
UK_Fet	46.0	29.5	99.3	33.9	13.7	60.9	8.0	4.5	19.5	3.5	1.86	12.4	0.015	0.009	0.031	0.664	0.41	1.99	0.64	0.422	4.96
UK_Fet	268.4	188.2	648.1	388.0	330.4	493.1	101.6	74.9	251.7	75.4	35.54	134.7	0.058	0.026	0.111	1.806	0.69	3.72	0.48	0.229	4.49
All popul	35.0	7.1	2220.8	15.8	0.0	156.6	4.8	1.2	129.6	1.4	0.002	45.0	0.016	0.000	0.212	0.547	0.14	32.60	0.39	0.003	9.20
All popul	161.1	42.7	2522.3	211.4	27.6	658.6	54.5	8.6	251.7	23.0	0.002	254.9	0.050	0.003	0.586	0.766	0.02	32.67	0.38	0.015	7.60

B) Additional single measurements of the elemental composition of other body parts of two male Stag Beetles from two populations.																					
Population acronym	Body part	Ca	Mg	K	Na	Mn	Fe	Zn	Cu	As	Cd	Pb	Ni								

POL_PrzB	head with mandibles	162.7	443.6	5017.1	715.4	20.3	156.6	87.7	3.96	1.91	0.02	0.47	0.33								
POL_PrzB	pronotum with legs	261.7	599.3	4850.7	948.8	22.3	218.8	108.2	4.62	1.35	0.02	0.90	0.71								
POL_Mś	wings	583.7	750.1	9635.1	1633.3	251.1	492.3	43.3	505.8	2.85	0.27	3.01	0.93								
POL_Mś	head with antenna	206.3	600.5	6407.8	1060.8	16.1	23.5	76.1	4.75	7.04	0.01	1.02	0.30								
POL_Mś	pronotum with legs	263.1	634.5	5344.5	965.3	18.1	23.1	77.1	4.70	0.76	0.01	1.76	0.96								
POL_Mś	mandibles	179.4	323.4	1318.4	529.3	22.1	5.9	6.3	9.03	1.22	0.02	0.14	0.09								
POL_Mś	abdomen with legs	217.9	1395.6	8428.0	1078.9	21.2	302.2	58.0	2.52	0.44	0.01	0.07	0.10								

Note: from two populations (GER_For, GER_Ta) only the elytra were sampled.

**Table 3:** Spearman rank correlation coefficients (r_s_) testing the relationship between the size of elytra and abdomens (= sample mass) and concentrations of 12 chemical elements measured in various Stag Beetle *Lucanus cervus* populations (see Fig. 1 and Table 1 for the origin of the populations) with sample sizes *n* > 10; the statistically significant relationships are in bold; * *P* ≤ 0.05, ** *P* < 0.01, *** *P* < 0.001.

Sample/population acronym	Number of samples	Ca	Mg	K	Na	Mn	Fe	Zn	Cu	As	Cd	Pb	Ni
**ELYTRA**													
All populations	271	−0.299***	−0.251***	0.109	0.195***	0.071	−0.281***	−0.214***	−0.350***	−0.262***	−0.461***	−0.478***	−0.175**
BEL_Sol	12	0.091	0.112	0.322	0.196	0.189	−0.014	−0.259	−0.336	0.126	−0.427	−0.503	−0.706**
BEL_Spe	33	−0.218	−0.274	−0.122	−0.079	−0.115	0.080	−0.050	−0.417*	−0.130	−0.412*	−0.164	−0.156
GER_Ko	11	0.382	0.173	0.209	0.164	−0.282	−0.336	−0.364	−0.555	−0.709*	−0.636*	−0.436	−0.227
GER_Ta	18	−0.172	−0.459	−0.020	0.267	−0.115	−0.366	0.121	−0.441	−0.156	0.117	−0.232	−0.521*
ITA	11	0.345	0.345	−0.555	−0.700*	−0.055	0.564	−0.500	−0.645*	−0.055	−0.318	−0.409	0.436
POL_Łsz	12	−0.049	−0.042	0.483	0.000	0.594*	0.350	0.161	−0.035	−0.252	0.007	−0.734**	−0.636*
POL_Prz	30	−0.459*	0.041	0.055	0.223	0.334	0.131	−0.365*	−0.107	−0.001	−0.503**	−0.310	−0.458*
POL_Wł	10	−0.912***	−0.505	−0.255	0.097	−0.918***	−0.894***	−0.243	−0.322	−0.723*	−0.365	−0.705*	−0.274
Slo_MS3	10	0.024	0.644*	0.413	0.298	0.973***	−0.347	0.480	−0.030	−0.681*	−0.328	−0.681*	0.407
SPA_La	17	−0.735***	−0.554*	0.294	0.098	0.017	−0.400	0.529*	−0.664**	−0.289	−0.127	−0.483*	−0.228
UK_Col	14	−0.714**	−0.490	−0.789***	−0.244	−0.516	−0.811***	−0.644*	−0.587*	−0.719**	−0.484	−0.732**	−0.226
UK_Fet	12	0.350	0.007	0.699*	0.524	0.070	0.811**	0.196	−0.203	0.874***	0.105	−0.168	0.469
**ABDOMENS**													
All populations	124	−0.312***	0.042	0.327***	0.295***	−0.004	−0.123	−0.355***	0.041	−0.127	−0.513***	−0.515***	−0.457***
BEL_Spe	19	−0.282	−0.212	0.226	0.116	0.142	0.516*	−0.075	−0.253	0.026	0.023	0.181	−0.325
POL_Prz	15	−0.425	0.129	0.232	0.407	−0.504	−0.429	−0.600*	0.454	0.604*	−0.357	−0.471	−0.404

**Additional comment on the data in**Table 3**.**

Given the apparent sexual dimorphism in body size in Stag Beetles, our findings suggest that sex-related differences in ETs for some of these metals can occur in this species. Sex-related differences in the contents of major nutrients and trace elements have been described in dimorphic insects, and it has been hypothesized that this can have certain consequences for their reproduction [15]. To date, given that female Stag Beetles are the smallest individuals [9; see Photo 1], we can speculate that they tend to accumulate relatively more Zn, As, Cd and Ni in both abdomens and elytra, and more Pb and Mg in their abdomens and elytra, respectively. At the same time, higher abdomen K concentrations may be indicative of larger males. These findings are therefore a clear incentive for further detailed assessments under controlled conditions of how ETs in different body parts of beetles and other insects vary with sex and body size heterogeneity – the rule in a population but often overlooked in the stoichiometry/elemental study of insects – and relating this to reproductive indices and landscape heterogeneity.

**Table 4:** Pearson correlation coefficients with original *P*-values testing the relationship between the mass and concentrations of elements measured in abdomens and elytra in 28 Stag Beetle *Lucanus cervus* populations (data average per population) and six major land-cover types determined within three radii of 500 m, 1000 m and 5000 m (for clarity, each radius is shown against a different background) around the sampling sites (i.e. exact population location; for details, see Table 1). * *P* ≤ 0.05, ** *P* < 0.01, *** *P* < 0.001; the results meeting the FDR-adjusted *P*-value are indicated in bold.

Element / body part	Radius	Urban/built-up area	Woods/ parks	Agriculturalland	Grassland/ open natural habitats	Roads/railways	Water
Abdomen mass	500 m	−0.517**	0.492*	−0.174	−0.046	−0.332	0.031
	1000 m	−0.382	0.492*	−0.056	−0.306	−0.294	0.047
	5000 m	−0.461*	0.361	0.159	−0.378	−0.439*	0.193
Elytra mass	500 m	−0.379	0.398*	−0.174	−0.131	−0.277	−0.122
	1000 m	−0.258	0.421*	−0.206	−0.296	−0.219	−0.041
	5000 m	−0.191	0.398*	−0.164	−0.133	−0.313	0.085
Abdomen Ca	500 m	0.697***	−0.605***	0.037	0.170	0.429*	0.252
	1000 m	0.557**	−0.510**	0.201	0.160	0.140	0.167
	5000 m	0.628***	−0.596**	0.265	0.172	0.244	0.087
Elytra Ca	500 m	0.221	−0.081	0.069	−0.048	−0.092	−0.172
	1000 m	0.123	−0.112	0.355	−0.254	−0.007	−0.005
	5000 m	0.217	−0.280	0.493	−0.440	−0.024	0.059
Abdomen Mg	500 m	0.460*	−0.323	−0.203	0.188	0.545**	0.262
	1000 m	0.381	−0.294	−0.132	0.254	0.369	0.061
	5000 m	0.361	−0.261	−0.151	0.165	0.411	−0.017
Elytra Mg	500 m	0.071	−0.192	−0.129	0.484*	0.154	−0.168
	1000 m	0.005	−0.236	−0.110	0.489*	0.113	−0.276
	5000 m	0.057	−0.462*	0.148	0.161	0.217	−0.131
Abdomen K	500 m	−0.056	0.153	−0.218	−0.023	0.247	−0.062
	1000 m	−0.071	0.172	−0.284	0.023	−0.020	−0.300
	5000 m	−0.004	0.002	−0.033	−0.024	−0.041	−0.190
Elytra K	500 m	−0.014	0.109	−0.191	−0.065	0.230	0.148
	1000 m	−0.026	0.164	−0.237	0.015	−0.069	−0.101
	5000 m	−0.003	0.056	−0.080	−0.022	−0.104	−0.111
Abdomen Na	500 m	−0.003	−0.033	0.043	0.055	0.366	0.048
	1000 m	0.069	−0.050	−0.040	0.104	0.021	−0.121
	5000 m	0.088	−0.173	0.033	0.165	0.082	−0.061
Elytra Na	500 m	−0.175	−0.112	0.237	0.203	0.098	−0.157
	1000 m	−0.128	−0.052	−0.028	0.219	−0.078	−0.241
	5000 m	−0.062	−0.282	0.152	0.219	0.008	−0.158
Abdomen Mn	500 m	−0.174	0.303	−0.324	−0.145	−0.049	0.055
	1000 m	−0.200	0.373	−0.359	−0.143	−0.209	−0.154
	5000 m	−0.185	0.411*	−0.312	−0.113	−0.294	−0.223
Elytra Mn	500 m	−0.305	0.483*	−0.417*	−0.173	−0.156	−0.207
	1000 m	−0.336	0.496	−0.360	−0.202	−0.181	−0.377
	5000 m	−0.315	0.445*	−0.129	−0.435*	−0.338	−0.286
Abdomen Fe	500 m	0.290	−0.259	−0.176	0.264	0.247	0.429*
	1000 m	0.272	−0.182	−0.194	0.300	−0.080	0.277
	5000 m	0.213	−0.030	−0.321	0.375	0.015	−0.006
Elytra Fe	500 m	0.254	−0.181	−0.049	0.059	0.040	0.103
	1000 m	0.251	−0.192	0.095	0.135	−0.122	0.026
	5000 m	0.091	−0.126	0.104	0.135	−0.095	−0.113
Abdomen Zn	500 m	0.536**	−0.358	−0.003	0.012	0.586**	−0.097
	1000 m	0.445*	−0.359	−0.097	0.222	0.305	−0.277
	5000 m	0.580**	−0.408*	−0.070	0.252	0.403*	−0.353
Elytra Zn	500 m	0.113	−0.077	−0.128	−0.003	0.152	0.407*
	1000 m	−0.011	0.016	−0.085	0.083	−0.327	0.153
	5000 m	−0.006	0.060	−0.083	0.119	−0.184	−0.045
Abdomen Cu	500 m	−0.015	0.039	−0.190	−0.004	0.380	0.307
	1000 m	0.001	0.072	−0.239	0.082	−0.001	0.090
	5000 m	0.062	−0.030	−0.110	0.077	0.062	0.267
Elytra Cu	500 m	0.241	−0.088	−0.101	−0.265	0.211	0.445*
	1000 m	0.147	0.005	−0.061	−0.018	−0.178	0.236
	5000 m	0.115	0.086	−0.037	−0.071	−0.127	−0.093
Abdomen As	500 m	0.102	−0.297	−0.058	0.428*	0.283	−0.056
	1000 m	0.070	−0.371	−0.137	0.531**	0.177	−0.054
	5000 m	0.159	−0.375	−0.270	0.424*	0.430*	0.221
Elytra As	500 m	0.190	−0.454*	−0.107	0.648***	0.398*	0.303
	1000 m	0.190	−0.442*	−0.328	0.639***	0.108	0.190
	5000 m	0.242	−0.336	−0.559**	0.636***	0.398*	0.165
Abdomen Cd	500 m	0.178	−0.195	0.291	−0.139	0.071	−0.283
	1000 m	0.019	−0.182	0.225	0.280	0.046	−0.301
	5000 m	0.079	−0.167	0.211	0.138	0.188	−0.394*
Elytra Cd	500 m	0.077	−0.010	−0.070	−0.080	−0.053	−0.199
	1000 m	−0.108	0.018	−0.087	0.192	−0.116	−0.315
	5000 m	−0.023	−0.120	0.191	−0.096	−0.024	−0.285
Abdomen Pb	500 m	0.615**	−0.370	−0.211	0.070	0.299	−0.048
	1000 m	0.437*	−0.263	−0.170	0.157	0.066	−0.115
	5000 m	0.508**	−0.196	−0.133	0.140	0.171	−0.294
Elytra Pb	500 m	0.003	0.142	−0.196	−0.099	−0.086	−0.109
	1000 m	−0.136	0.177	−0.032	−0.128	−0.169	−0.198
	5000 m	−0.020	−0.078	0.346	−0.334	−0.166	0.167
Abdomen Ni	500 m	0.155	0.056	−0.041	−0.332	0.068	0.022
	1000 m	0.104	0.083	−0.011	−0.197	−0.133	−0.081
	5000 m	0.119	0.193	−0.122	−0.017	−0.130	−0.358
Elytra Ni	500 m	0.234	−0.211	0.172	−0.103	0.075	0.202
	1000 m	0.211	−0.233	0.229	−0.091	−0.053	0.242
	5000 m	0.125	−0.126	0.070	0.046	0.013	0.032

## 2 Experimental design, materials and method

### 2.1 Species and populations

This data applies to adult Stag Beetles collected at 28 localities (hereafter referred to as 'local populations') situated in eight European countries ranging from Central Spain to Western Russia (Fig. 1; for georeferences, see Table 1).  Due to the scarcity of their primary larval food source, i.e. a lack of dead wood resources, Stag Beetles, in common with the community of saproxylic invertebrates as a whole, are an endangered species and have, owing to their iconic status, been identified as good indicators of woodland quality (see [2]).

Stag Beetles are known to have been present for many decades in some of the regions covered by this data article, for example, Belgium [2], Germany [3], the UK [4], Italy [5] and Poland (D. Tarnawski, A. Smolis, M. Kadej – unpubl. results). Furthermore, some of the Stag Beetle populations included in this study have already been extensively examined, from a biometric standpoint (data from several local European populations collated in [4, 9]), pattern of distribution (Belgium: [2]), movement (telemetry) of individuals (Germany: [3]; Italy: [5]), mating behaviour [6] and a pan-European monitoring programme [7].

The beetles used in this data article were already dead and were acquired from relatively small areas (up to *c.* 100 m in diameter); they included roadkill victims, predatory remains or those dead from unknown causes, most probably natural death after mating (see Table 1). All the Stag Beetles were collected by researchers very familiar with the appearance of this species, so confusion with other species of large beetles can be ruled out. Stag Beetles from most of the populations examined here, formed the basis of a recent study on the genetic diversity of this species in Europe, and many of the individual beetles were from an existing collection [8]. In addition, although there are four other members of genus *Lucanus* in Europe – *L. (Pseudolucanus) barbarossa, L. pontbrianti, L. tetraodon* and *L. ibericus* – the materials used for this article were collected outside the geographical ranges of those species.

For the analysis we used intact abdomens (*n* = 124) with dry internal organs and gut contents, and both elytra (*n* = 271); other parts (legs) of the majority of these beetles were also used in the genetic study by Cox et al. [8]. The samples were dried and kept at room temperature until the chemical analysis.

Female Stag Beetles more often move around only on the ground and are less capable of dispersing, and they often succumb to predators [3, 4, 9].

Since in most cases only fragments of the beetles were available, reliable sexing of all of them was impossible; for this reason we did not analyse sex-related differences. However, taking into account the large differences in elytra masses (a function of beetle size) measured in individual populations (up to 2.7-fold between extreme values, as in the Przemków forest, Poland; see Fig. 1), our samples may contain remains of both sexes. Even though Stag Beetles demonstrate a normal size distribution [4], male Stag Beetles in Europe can be up to *c.* 30% larger than females (see Photo 1), and even among males nearly 3-fold differences in body length can occur [9].

### 2.2 Land-cover types inhabited by the individual populations

For each sampling site, defined here as the central point of the sampling area, we measured the areas (ha) of several land-cover classes lying within three radii of that point (500 m, 1000 m and 5000 m) using Arc-View software from satellite imagery maps taken from Google Maps and other supporting information sources of local land-use types. These classes were ultimately pooled into six major land-cover types based primarily on the ability of Stag Beetles to utilize them: urban (different types of built-up areas, including industrial buildings); wooded (woods, parks and forests); agricultural (arable land; croplands and different cultivations); grassland (meadows, pastures and other open habitats with low vegetation, such as steppe-like habitats in Spain and Russia); transport infrastructure (tarred roads and highways, and railways); water (lakes, gravel pits and rivers). We assessed the effects of these six major land-cover types on the elytra and abdomen masses (hereafter referred to simply as 'body size') and ETs of Stag Beetles between successive radii. Generally, we did not prioritize any of the land-cover types or radii, because each could be regarded as causing variation in the body size and ETs of Stag Beetles. However, with a knowledge of Stag Beetle biology and ecology, one can make a number of predictions. First of all, deciduous woodland is the key land-cover type (resource) for the persistence of a Stag Beetle population in mainland Europe [2], though not in the UK, where the overwhelming majority of populations live in anthropogenic habitats, which suggests their opportunistic/synanthropic occurrence [4, 9]. Secondly, these beetles are poor dispersers: the home ranges of both sexes are very small, and adults do not move more than 500 m from their reproduction sites [3, 5]. More importantly, we assumed that because Stag Beetles do not need to feed in the adult stage, the quality of the larval habitat (the amount of coverage of wooded area representing the availability of dead wood) may be crucial in explaining their elemental traits and size (this latter point is discussed in [4, 9]). Consequently, Stag Beetles living in poor-quality habitats, i.e. urban areas with small percentages of wooded areas where natural decaying wood is scarce and larvae often feed on various anthropogenic woody substitutes, such as railway sleepers, fence posts and the like in urban gardens, as in the UK, may be smaller in size than those from extensive woodlands (cf. [9]). Thus, we expected that the contribution of two major land-cover types (urban and wooded, representing a negative and a positive influence, respectively), especially in the smallest, 500 m radius, would be stronger than in the other two, larger radii (1000 m and 5000 m). These latter two radii may represent the scale of spatial drift or bioaccumulation into topsoil of metallic pollutants, such as agrochemicals (from crop fields/cultivations or recreational areas) or traffic-related metals (from transport infrastructure and vehicle emissions). Importantly, since railway sleepers are impregnated with creosote, they are sources of both inorganic and organic contaminants that are accumulated in local food webs [10]. Data from the UK suggest, however, that Stag Beetles rarely use railway sleepers [11].

### 2.3 Chemical analysis

Most of the samples showed no signs of adhering extraneous material; examined under the binocular microscope, the smooth chitin surfaces of the inner and outer layers of the elytra and the abdominal segment surfaces were clean. Nevertheless, the samples were thoroughly cleaned (dusted) with a stream of air from a rubber blower. Any impurities like sand grains, fragments of plant tissue, leaf litter or streaks of dirt on the chitin surfaces were also removed mechanically with a soft plastic bristle brush and blown clean with a stream of air. The samples were then dried at 50 °C to constant weight, and weighed using a digital analytical balance (ONYX-220 FAWAG) with ± 0.0001 g precision.

The entire elytra and abdomens (0.0159 – 0.312 g of dry weight) were digested in 3 ml nitric acid (ultrapure, 65%, Merck) and 0.5 ml perchloric acid (ultrapure, 62%, Merck) in a CEM Mars 5 Corporation microwave oven [12]. The digestion procedure was conducted in three temperature steps (110 °C/10 min, 185 °C/25 min, 200 °C/15 min) at a maximum pressure of 200 psi. After dilution to 10 ml, the digests were analysed for Ca, Fe, K, Mg, Mn, Na and Zn using FAAS and an AVANTA PM GBC Atomic Absorption Spectrophotometer (Ca, K, Na in the emission mode of the spectrophotometer), and As, Cd, Cu, Ni and Pb using ETAAS with a Graphite Furnace and a Perkin-Elmer PinAAcle 900Z Atomic Absorption Spectrophotometer [13]. All the elements were determined against standards (Atomic Absorption Standard Solutions from Sigma Chemical Co.) and blanks containing the same matrix as the samples and were subjected to the same procedure. All the results for the abdomens and elytra were calculated on a dry weight basis. The accuracy of the methods applied for the determination of the elements in the samples was checked by the analysis of Certified Reference Materials. We used DC73348 LGC standards of bush branches and leaves as certified reference materials. The recovery rates were from 94% to 103%.

### 2.4 Statistical analysis

The statistical analysis involved four major stages of data exploration. Initially, we compared the masses of elytra and abdomens and the metal concentrations (in ppm d.w.) measured in both body parts across the studied populations. As we found substantial variation in both masses and ETs between elytra and abdomens (see Results in [1]), the ETs of each of these two body parts were analysed separately.

We used a general linear model (in Statistica 12; Data Analysis Software System, Version 12.1. Tulsa: USA) to assess whether elytra and abdomen ETs were related to the masses of these body parts (see Results in [1]). Owing to the large differences in ETs amongst populations, we repeated this analysis using the Spearman rank correlation coefficients to test the relationship between the sample mass and elytra, and abdomen metal concentrations within individual populations with sample sizes *n* > 10 (Table 3).

We used Pearson correlation coefficients to assess the influence of local major land-cover types measured within three radii (500 m, 1000 m and 5000 m) around the sampling sites on the masses and elemental traits of abdomens and elytra (Table 4). Owing to the large number of tests within a particular radius (*n* = 6), we applied the false discovery rate (FDR) procedure to adjust the original *P*-values using the classical one-stage method in the statistical software spreadsheet [14]. To meet the assumption of normality (assessed by the Kolmogorov-Smirnov test) required for linear modelling, we log-transformed the following variables before the analysis: elytra and abdomen Pb concentrations; coverage of built-up areas, agricultural land, grassland, transport infrastructure and water measured within 500 m; coverage of built-up areas, grassland and water measured within 1000 m; and grassland, transport infrastructure and water measured within 5000 m.

## Declaration of Competing Interest

The authors declared they do not have anything to disclose regarding conflict of interest with respect to this manuscript.

## Figures and Tables

**Table:** 

Country	Population (acronym)	Body part	Sample mass (g)	Ca ppm	Mg ppm	K ppm	Na ppm	Mn ppm	Fe ppm	Zn ppm	Cu ppm	As ppm	Cd ppm	Pb ppm	Ni ppm
BEL	BEL_Dro	abdomen	0.0737	812.6	3268.8	6123.4	713.70	13.98	153.5	156.30	18.63	2.676	0.018	0.5716	0.419
BEL	BEL_Dro	abdomen	0.1149	465.6	5342.7	9135.3	1043.43	9.16	156.7	170.56	27.36	2.737	0.019	1.1652	0.639
BEL	BEL_Dro	abdomen	0.0657	984.7	5537.5	7085.7	707.00	17.73	323.1	210.85	12.47	16.932	0.094	0.5467	0.217
BEL	BEL_Dro	elytra	0.0524	291.3	889.0	1455.3	265.27	7.69	33.8	16.16	4.06	0.677	0.007	0.2338	0.044
BEL	BEL_Dro	elytra	0.0516	185.4	584.7	1133.2	215.89	5.64	24.6	0.00	3.17	0.316	0.005	0.3393	0.088
BEL	BEL_Dro	elytra	0.0525	468.7	597.9	2160.2	274.86	5.12	24.0	11.06	3.42	0.330	0.004	0.3643	0.114
BEL	BEL_Dro	elytra	0.0462	373.1	455.1	2039.5	277.27	7.03	17.3	6.02	3.77	0.242	0.007	0.3945	0.194
BEL	BEL_Dro	elytra	0.0370	264.6	613.0	2293.6	419.19	6.36	48.6	38.59	4.52	2.212	0.022	1.7233	0.154
BEL	BEL_Dro	elytra	0.0364	426.8	514.3	1977.1	442.86	7.69	52.7	31.58	4.54	1.645	0.012	1.2455	0.245
BEL	BEL_Dro	elytra	0.0313	516.7	591.2	2151.9	272.52	5.01	43.8	11.21	4.29	0.673	0.011	0.6654	0.315
BEL	BEL_Dro	elytra	0.0426	383.2	594.9	2096.8	247.42	6.84	25.4	10.79	3.98	0.612	0.009	0.3749	0.200
BEL	BEL_Ov	elytra	0.0301	330.4	514.3	2344.8	891.03	5.21	194.0	53.47	7.97	4.305	0.046	1.3242	9.197
BEL	BEL_Ov	elytra	0.0310	575.1	627.1	2847.4	1288.71	2.89	31.9	49.57	7.43	3.731	0.024	0.9790	4.840
BEL	BEL_Ov	elytra	0.0310	317.3	311.2	1186.4	230.97	6.50	57.1	5.85	7.46	3.878	0.024	0.8013	1.999
BEL	BEL_Ov	elytra	0.0375	1765.0	361.0	784.2	198.40	23.89	966.7	54.53	9.49	0.941	0.082	3.5839	5.165
BEL	BEL_Ov	abdomen	0.0464	567.2	2928.1	9862.1	1776.94	19.31	221.1	547.75	58.84	51.145	0.059	1.8007	1.782
BEL	BEL_Ov	abdomen	0.0337	1294.5	1313.9	1816.1	353.12	21.27	868.2	96.58	13.15	17.248	0.054	1.4783	2.621
BEL	BEL_Sol	elytra	0.0415	867.0	348.7	856.1	119.76	60.72	1043.1	37.90	6.90	1.464	0.123	4.9231	1.680
BEL	BEL_Sol	elytra	0.0389	1175.8	771.8	998.9	143.70	96.16	2220.8	43.55	5.98	1.796	0.171	9.7953	2.490
BEL	BEL_Sol	elytra	0.0306	509.7	397.6	950.8	221.90	13.54	154.9	15.82	12.04	0.988	0.038	2.1829	3.493
BEL	BEL_Sol	elytra	0.0324	510.6	555.6	948.1	217.59	12.10	113.0	12.32	10.37	1.242	0.028	1.1185	3.076
BEL	BEL_Sol	elytra	0.0405	332.4	544.0	2324.0	282.47	17.70	132.1	25.10	17.06	0.807	0.028	1.4729	1.166
BEL	BEL_Sol	elytra	0.0403	343.7	586.0	2273.9	277.42	12.78	48.6	18.32	6.49	0.679	0.017	0.5585	2.160
BEL	BEL_Sol	elytra	0.0598	290.2	447.9	2683.9	318.06	6.37	25.1	8.09	4.83	0.711	0.016	0.4341	0.991
BEL	BEL_Sol	abdomen	0.0693	680.9	2580.8	7658.4	650.94	22.46	327.0	348.92	95.65	43.313	0.138	32.1371	0.904
BEL	BEL_Sol	abdomen	0.0700	2509.7	3323.3	6349.6	554.29	22.72	943.6	307.15	30.24	15.232	0.046	1.5826	0.651
BEL	BEL_Sol	abdomen	0.0756	664.9	3118.1	5873.4	642.59	12.15	151.9	161.17	21.34	5.803	0.046	0.8448	0.499
BEL	BEL_Sol	abdomen	0.0780	1628.2	8412.9	7426.5	687.95	22.40	354.5	210.41	44.67	87.951	0.058	1.8548	0.459
BEL	BEL_Sol	abdomen	0.0675	1020.1	10,347.7	6432.0	612.15	23.89	237.6	269.30	22.71	23.488	0.040	11.4131	0.339
BEL	BEL_Sol	abdomen	0.0249	2158.9	809.6	945.5	294.38	31.04	146.2	97.19	41.53	2.257	0.090	1.3601	0.902
BEL	BEL_Sol	elytra	0.0455	1410.1	1099.8	1772.9	225.93	19.94	138.9	10.90	7.57	3.975	0.014	0.8394	0.551
BEL	BEL_Sol	elytra	0.0484	2672.5	2036.5	2432.4	269.42	22.91	199.8	33.50	9.04	5.745	0.023	0.9722	0.417
BEL	BEL_Sol	elytra	0.0408	381.2	545.3	2098.2	431.86	16.47	24.5	6.23	4.48	1.948	0.008	1.1094	0.358
BEL	BEL_Sol	elytra	0.0409	293.1	568.6	2067.0	447.19	16.98	7.1	2.96	3.54	1.799	0.006	0.9755	0.462
BEL	BEL_Sol	elytra	0.0480	547.9	553.5	661.1	170.42	19.83	251.5	9.58	6.21	0.389	0.018	1.3017	0.674
BEL	BEL_Spe	elytra	0.0386	395.7	548.4	3815.5	496.89	17.70	20.7	55.17	9.13	0.734	0.067	0.8843	0.316
BEL	BEL_Spe	elytra	0.0396	368.9	494.5	3278.2	421.72	14.99	22.7	27.50	6.86	0.496	0.026	0.8624	4.548
BEL	BEL_Spe	elytra	0.0240	465.8	654.0	3879.4	630.00	37.80	70.4	16.64	8.65	2.493	0.038	7.8629	0.797
BEL	BEL_Spe	elytra	0.0267	683.6	1307.9	4925.9	570.41	33.56	44.2	35.80	15.56	3.423	0.094	0.4399	0.330
BEL	BEL_Spe	elytra	0.0509	257.8	333.8	2805.9	319.45	13.64	18.1	7.13	4.45	1.772	0.032	0.5059	0.360
BEL	BEL_Spe	elytra	0.0508	394.5	732.8	2466.7	292.91	13.23	31.1	10.72	8.10	1.660	0.017	0.2685	0.218
BEL	BEL_Spe	elytra	0.0336	495.3	540.0	3048.0	402.38	8.33	17.0	12.60	8.60	1.025	0.030	0.4836	0.372
BEL	BEL_Spe	elytra	0.0341	584.1	568.0	3113.1	410.56	13.14	58.4	13.84	10.74	1.433	0.031	6.6728	0.330
BEL	BEL_Spe	elytra	0.0492	429.1	638.0	2628.8	391.87	10.47	29.3	12.05	7.32	0.295	0.022	0.5599	0.198
BEL	BEL_Spe	elytra	0.0486	2299.8	616.3	747.9	142.59	21.66	357.6	14.69	8.66	0.582	0.075	1.6379	0.761
BEL	BEL_Spe	elytra	0.0384	3629.5	1273.1	1274.9	179.43	44.63	1141.9	73.42	9.24	0.209	0.087	4.0011	1.217
BEL	BEL_Spe	elytra	0.0427	1496.5	664.4	922.5	184.31	27.28	44.5	13.32	4.70	0.371	0.039	0.4578	0.315
BEL	BEL_Spe	elytra	0.0479	323.4	517.1	2548.2	243.42	15.67	19.2	11.37	3.96	2.400	0.021	0.2677	0.075
BEL	BEL_Spe	elytra	0.0332	383.6	585.5	2854.1	475.30	14.17	20.5	17.13	4.88	0.895	0.026	0.4426	0.148
BEL	BEL_Spe	abdomen	0.0762	696.6	2275.3	5916.5	794.09	22.19	99.7	184.98	11.52	1.549	0.043	1.2270	0.407
BEL	BEL_Spe	abdomen	0.1017	575.2	9611.3	9674.1	1011.60	18.94	136.8	184.64	23.48	35.748	0.094	1.1794	0.193
BEL	BEL_Spe	abdomen	0.0470	782.6	2037.4	7633.2	911.28	16.68	86.4	287.98	20.76	51.817	0.112	0.6688	0.312
BEL	BEL_Spe	abdomen	0.0607	525.2	2817.1	6395.1	544.48	28.42	79.2	128.77	10.50	3.951	0.158	1.8839	1.053
BEL	BEL_Spe	abdomen	0.0384	620.2	1681.9	6458.7	848.44	17.21	65.9	205.76	21.66	11.473	0.112	0.9909	1.873
BEL	BEL_Spe	abdomen	0.0441	614.5	4759.2	9743.8	1282.99	31.24	118.8	525.97	68.69	186.893	0.081	1.1138	0.287
BEL	BEL_Spe	abdomen	0.0410	734.9	2339.1	6447.7	766.34	17.76	45.9	305.75	156.07	17.457	0.370	0.7766	0.274
BEL	BEL_Spe	abdomen	0.0521	669.9	5287.4	6584.6	969.87	16.55	158.3	250.13	43.95	149.820	0.080	1.2084	1.072
BEL	BEL_Spe	abdomen	0.0263	685.8	2652.8	3247.0	460.08	19.59	92.4	149.06	84.76	60.236	0.055	1.1171	1.555
BEL	BEL_Spe	abdomen	0.0597	676.4	2782.3	7376.9	966.33	20.26	103.2	301.77	28.56	22.531	0.067	0.8063	0.289
BEL	BEL_Spe	abdomen	0.0585	511.5	3121.2	4160.4	457.95	26.23	202.7	126.17	17.02	1.936	0.044	0.9852	0.421
BEL	BEL_Spe	abdomen	0.0377	789.2	4031.6	4451.4	752.79	29.71	53.3	184.87	24.05	7.568	0.048	1.2062	7.601
BEL	BEL_Spe	elytra	0.0442	415.6	581.5	3005.6	425.34	13.18	37.8	19.16	5.99	1.498	0.010	0.7090	0.542
BEL	BEL_Spe	elytra	0.0419	447.9	546.4	2773.4	436.52	13.63	13.4	7.80	4.94	1.014	0.011	0.8850	0.283
BEL	BEL_Spe	elytra	0.0329	583.8	763.8	1704.2	373.25	21.79	16.7	9.19	5.41	1.003	0.013	1.0919	6.290
BEL	BEL_Spe	elytra	0.0351	647.9	1046.2	1839.3	375.21	20.74	32.2	22.06	5.68	1.514	0.016	0.5765	0.257
BEL	BEL_Spe	elytra	0.0509	1220.6	441.3	658.7	304.91	20.46	60.5	25.91	4.08	3.577	0.026	0.7442	0.996
BEL	BEL_Spe	elytra	0.0276	437.0	560.9	1349.5	255.07	11.77	43.8	14.47	5.53	8.742	0.035	2.0585	3.149
BEL	BEL_Spe	elytra	0.0294	1690.7	391.8	496.6	242.52	18.67	53.7	0.00	4.99	0.657	0.046	0.8961	0.548
NET	BEL_Spe	abdomen	0.0626	590.5	1802.3	5082.0	731.31	21.65	192.3	262.42	37.01	62.953	0.066	0.7313	0.811
NET	BEL_Spe	abdomen	0.1361	2940.5	1514.1	4457.3	764.88	62.38	1243.1	110.74	15.64	31.322	0.069	3.9484	2.180
NET	BEL_Spe	elytra	0.0485	855.3	570.1	2365.7	428.45	22.63	434.0	22.95	5.72	3.460	0.029	1.6355	3.369
NET	BEL_Spe	elytra	0.0437	618.1	378.9	2224.9	428.38	19.73	186.5	18.27	5.30	2.955	0.026	1.1375	2.723
NET	BEL_Spe	elytra	0.0463	451.4	489.8	2352.1	414.04	13.06	59.4	17.51	5.63	4.517	0.012	0.6136	1.118
NET	BEL_Spe	elytra	0.0461	353.8	465.4	2248.6	411.93	10.93	54.9	16.27	4.58	4.214	0.009	0.7141	1.288
NET	BEL_Spe	elytra	0.0459	409.6	409.4	1780.6	361.22	11.71	134.2	12.13	4.57	2.526	0.016	0.9207	0.565
NET	BEL_Spe	elytra	0.0558	476.5	574.2	2291.3	362.01	21.08	260.8	16.91	3.87	0.684	0.020	0.9142	0.648
BEL	BEL_Spe	abdomen	0.0695	445.2	2053.3	9181.9	1227.05	16.28	90.8	326.44	52.18	25.553	0.049	0.8906	0.150
BEL	BEL_Spe	abdomen	0.0365	637.2	4998.6	7371.5	1048.77	13.81	77.5	218.46	10.83	10.364	0.030	0.7027	0.595
BEL	BEL_Spe	abdomen	0.0456	796.0	3314.2	4095.9	775.88	17.19	160.5	225.81	45.79	16.071	0.049	1.9196	0.615
BEL	BEL_Spe	abdomen	0.0478	752.8	3850.0	6913.4	821.13	11.95	161.1	166.31	29.09	9.129	0.031	0.7528	0.288
BEL	BEL_Spe	abdomen	0.0673	552.2	3179.6	7953.2	1185.29	12.32	113.1	353.76	56.18	32.645	0.052	0.5510	0.211
BEL	BEL_Spe	elytra	0.0511	314.4	674.9	2894.9	599.61	10.08	11.7	8.76	3.78	0.515	0.008	0.7306	0.106
BEL	BEL_Spe	elytra	0.0499	966.2	600.2	2753.7	593.79	11.22	13.2	2.91	3.89	0.387	0.010	0.2294	0.104
BEL	BEL_Spe	elytra	0.0466	550.0	491.3	2470.3	425.11	11.30	26.4	14.54	4.26	0.822	0.010	0.2659	0.276
BEL	BEL_Spe	elytra	0.0447	283.5	528.3	1976.2	397.99	9.27	26.0	10.56	3.84	0.650	0.011	0.2868	0.088
BEL	BEL_Spe	elytra	0.0308	540.0	731.7	2485.2	366.23	6.18	16.9	1.18	4.04	1.816	0.011	0.3816	0.468
BEL	BEL_Spe	elytra	0.0313	508.9	736.1	2359.9	353.67	6.44	12.8	6.57	4.47	1.404	0.042	0.2292	0.076
GER	GER_Afa	abdomen	0.1158	487.2	1920.0	11,062.7	1744.21	54.94	841.5	217.88	96.13	18.044	0.031	0.9314	1.405
GER	GER_Afa	abdomen	0.1978	364.2	3983.3	10,293.7	2243.17	74.46	783.3	123.92	21.69	1.092	0.021	1.4337	1.412
GER	GER_Afa	elytra	0.0590	200.4	700.5	5297.6	728.47	48.79	1075.1	54.35	6.92	2.862	0.019	1.4545	1.815
GER	GER_Afa	elytra	0.0667	227.6	641.2	5828.2	777.66	39.63	502.7	48.62	6.45	2.743	0.016	0.8211	1.139
GER	GER_Afa	elytra	0.0702	481.9	777.4	2070.6	795.44	29.04	131.9	11.20	6.90	1.714	0.014	0.3931	0.957
GER	GER_Afa	elytra	0.0698	377.0	683.9	2538.0	646.13	26.64	69.6	8.84	5.36	1.517	0.010	0.3429	1.273
GER	GER_Aho	abdomen	0.1346	483.3	4175.6	6214.7	986.78	35.36	297.1	119.00	20.24	1.272	0.017	0.2228	0.307
GER	GER_Aho	elytra	0.0608	353.8	491.4	2997.1	523.03	26.71	38.0	6.97	3.71	0.732	0.012	0.4335	0.461
GER	GER_Aho	elytra	0.0599	351.9	417.1	2743.0	528.71	27.86	28.9	5.45	3.81	0.605	0.006	0.1430	0.172
GER	GER_Aho	elytra	0.0642	362.0	661.7	2765.4	472.90	21.63	64.2	6.41	4.78	0.913	0.013	0.3549	0.428
GER	GER_Aho	elytra	0.0591	367.8	593.3	2704.0	423.69	19.71	50.1	5.73	4.09	0.943	0.014	0.2435	0.222
GER	GER_Bu	abdomen	0.1899	1006.0	8016.3	11,175.9	1949.13	49.66	668.1	119.67	136.44	0.093	0.017	1.7435	0.802
GER	GER_Bu	abdomen	0.0781	1100.4	5888.1	15,927.7	2520.87	85.61	1125.7	302.07	106.47	38.489	0.048	2.1010	1.241
GER	GER_Bu	abdomen	0.1117	1662.5	10,211.5	11,909.8	2572.85	64.17	167.0	193.11	219.07	18.498	0.007	0.3867	0.172
GER	GER_Bu	elytra	0.0507	411.1	1050.9	4539.5	804.34	21.43	73.4	20.76	10.48	2.367	0.013	0.7119	0.637
GER	GER_Bu	elytra	0.0513	409.5	962.8	4326.2	772.32	22.92	116.2	20.99	10.43	2.284	0.018	0.4553	1.485
GER	GER_Bu	elytra	0.0452	420.9	807.6	4430.4	755.09	24.28	303.1	34.00	12.13	3.835	0.013	0.7611	1.023
GER	GER_Bu	elytra	0.0476	378.3	658.0	5825.1	994.75	16.94	128.4	51.09	16.80	5.146	0.014	0.4411	1.036
GER	GER_Bu	elytra	0.0466	286.6	471.2	7145.5	1019.96	17.55	75.8	54.01	7.19	8.488	0.018	0.7240	1.033
GER	GER_Bu	elytra	0.0451	322.7	478.9	7253.5	1022.17	20.61	77.2	46.41	8.03	8.080	0.013	3.7934	0.572
GER	GER_For	elytra	0.0971	335.6	637.0	3189.6	1103.91	20.53	24.8	22.56	3.11	0.451	0.003	0.4678	0.325
GER	GER_For	elytra	0.0681	440.0	292.9	2848.7	594.86	12.33	65.1	37.85	2.91	0.939	0.007	0.6136	0.298
GER	GER_For	elytra	0.0634	394.6	353.2	3777.8	827.13	13.07	31.9	53.44	3.20	1.288	0.004	0.7238	1.300
GER	GER_For	elytra	0.0679	588.8	409.3	3594.4	827.98	9.90	72.0	46.15	3.36	0.979	0.006	0.9388	0.458
GER	GER_For	elytra	0.0496	440.4	480.5	2595.0	768.55	10.61	18.3	17.08	3.58	0.578	0.005	0.4191	0.154
GER	GER_For	elytra	0.0482	356.3	455.6	3314.7	784.44	10.46	14.7	13.30	3.57	0.825	0.004	0.2690	0.158
GER	GER_Ko	abdomen	0.1227	496.5	2106.6	8984.9	1489.89	18.35	129.3	181.77	53.42	13.056	0.016	0.2129	0.150
GER	GER_Ko	abdomen	0.0635	387.1	2994.5	4454.2	1008.98	25.57	215.6	91.66	59.51	59.365	0.065	0.4553	0.414
GER	GER_Ko	abdomen	0.0585	670.3	8601.8	11,190.2	1798.12	32.55	76.9	312.75	125.35	7.577	0.039	0.1806	0.056
GER	GER_Ko	abdomen	0.0552	720.8	3420.0	11,908.2	1586.96	18.87	117.2	407.20	81.25	30.327	0.040	1.0512	0.401
GER	GER_Ko	elytra	0.0499	412.5	686.8	3245.8	1066.33	16.83	18.0	6.79	3.15	0.681	0.007	0.2205	0.169
GER	GER_Ko	elytra	0.0802	334.2	392.3	2861.3	486.41	16.76	13.6	3.32	3.02	0.230	0.004	0.1496	0.567
GER	GER_Ko	elytra	0.0434	319.8	673.5	2317.8	532.03	24.26	36.6	22.86	5.35	1.660	0.014	0.2892	0.981
GER	GER_Ko	elytra	0.0435	298.8	579.3	2580.9	619.08	22.66	26.9	46.73	5.24	3.637	0.018	1.1859	0.294
GER	GER_Ko	elytra	0.0468	301.3	832.3	1336.7	662.61	20.58	94.0	8.27	8.77	11.198	0.026	0.3795	0.251
GER	GER_Ko	elytra	0.0480	295.1	820.5	1443.5	683.33	25.20	102.3	9.83	6.30	11.561	0.027	0.4705	0.897
GER	GER_Ko	elytra	0.0544	359.2	767.6	3170.4	750.55	22.85	36.2	20.02	4.67	1.081	0.008	0.2983	0.321
GER	GER_Ko	elytra	0.0518	405.9	704.7	3836.8	780.12	22.92	40.9	17.29	5.33	1.289	0.011	0.2686	0.177
GER	GER_Ko	elytra	0.0539	386.5	733.4	1075.7	782.37	12.05	19.9	1.57	3.53	1.343	0.010	0.2690	0.206
GER	GER_Ko	elytra	0.0534	482.5	926.3	1341.1	1026.78	13.63	21.0	2.04	4.38	1.394	0.017	0.2301	0.216
GER	GER_Ko	elytra	0.0449	394.7	699.2	1145.4	796.44	9.48	15.6	0.81	4.25	3.265	0.013	0.2236	0.261
GER	GER_Kro	abdomen	0.1100	676.1	1551.9	8167.2	2003.00	17.31	67.3	160.97	43.23	26.720	0.027	0.2730	0.224
GER	GER_Kro	abdomen	0.1780	733.1	2765.9	9797.1	2159.49	15.92	177.8	145.16	55.59	1.599	0.006	0.2016	0.239
GER	GER_Kro	elytra	0.0748	376.2	693.0	4358.5	1930.61	14.37	31.3	17.47	5.72	0.664	0.003	0.4126	0.527
GER	GER_Kro	elytra	0.0760	405.1	643.3	4188.4	1770.39	12.53	23.7	16.56	4.71	0.695	0.004	0.7622	0.454
GER	GER_Kro	elytra	0.0730	405.4	396.5	3762.3	763.15	10.89	25.3	8.62	3.65	0.838	0.004	0.3213	0.388
GER	GER_Kro	elytra	0.0752	384.9	368.6	3915.9	757.45	12.66	25.5	8.69	4.12	0.508	0.006	0.3864	0.666
GER	GER_Kro	elytra	0.0708	1573.6	1012.9	2264.9	748.16	20.25	121.5	8.55	4.74	0.571	0.006	0.5488	0.664
GER	GER_Kro	elytra	0.0730	1274.4	887.7	2032.3	757.95	18.72	81.5	6.13	4.72	0.583	0.006	0.4728	0.861
GER	GER_Kro	elytra	0.0617	600.1	734.0	2777.4	1197.08	12.89	66.6	6.08	3.53	1.570	0.005	0.5338	0.499
GER	GER_Kro	elytra	0.0521	474.9	592.9	2410.9	875.62	12.68	35.1	6.27	4.33	2.424	0.011	1.2306	0.389
GER	GER_Kro	elytra	0.0449	1266.4	954.1	3218.7	1284.19	23.45	72.6	4.04	4.72	2.288	0.006	0.7116	0.370
GER	GER_Sch	abdomen	0.0942	639.6	9230.8	14,302.5	2154.25	24.73	221.0	271.59	177.98	137.706	0.137	1.2852	0.510
GER	GER_Sch	abdomen	0.0868	710.8	2612.1	6717.5	1401.96	50.71	319.5	223.39	51.78	57.442	0.059	0.6377	0.767
GER	GER_Sch	elytra	0.0656	398.1	666.2	2873.9	1300.91	15.02	16.3	2.03	3.60	0.413	0.013	0.2708	0.524
GER	GER_Sch	elytra	0.0637	365.7	576.5	2539.6	1118.21	14.95	13.2	0.19	3.09	0.348	0.012	0.1452	0.411
GER	GER_Sch	elytra	0.0519	407.7	819.9	3074.9	944.70	24.82	19.7	8.63	5.50	5.376	0.017	0.5831	0.751
GER	GER_Sch	elytra	0.0547	338.0	643.7	2654.7	799.27	21.50	29.6	9.07	6.85	5.207	0.020	0.3374	1.045
GER	GER_Sch	elytra	0.0555	347.0	699.2	3877.3	717.30	11.10	59.8	22.02	7.61	5.618	0.017	0.4357	0.558
GER	GER_Sch	elytra	0.0584	354.6	588.1	2820.8	622.77	9.78	31.5	13.26	6.23	3.887	0.019	0.5667	0.389
GER	GER_Ta	elytra	0.0458	380.8	974.7	5686.5	1101.31	18.59	17.7	32.23	5.67	2.484	0.008	0.5104	1.450
GER	GER_Ta	elytra	0.0604	265.1	528.1	4237.1	821.69	19.84	11.8	17.03	3.83	1.486	0.008	0.3178	0.101
GER	GER_Ta	elytra	0.0568	360.6	595.8	3316.4	1237.32	14.79	13.2	2.13	3.63	2.833	0.005	0.4975	0.400
GER	GER_Ta	elytra	0.0586	333.9	554.1	3829.0	1166.89	13.57	14.5	7.85	3.63	3.090	0.005	0.3499	0.216
GER	GER_Ta	elytra	0.0796	356.2	500.2	1843.4	719.85	14.49	12.8	3.95	3.06	0.337	0.007	0.2561	0.173
GER	GER_Ta	elytra	0.0620	366.2	516.8	1683.5	782.26	10.66	17.7	1.17	2.75	0.219	0.007	0.3337	0.289
GER	GER_Ta	elytra	0.0443	487.0	464.8	808.8	335.21	6.57	30.9	0.27	3.23	0.400	0.012	0.4410	0.469
GER	GER_Ta	elytra	0.0503	959.6	765.8	1912.0	349.50	14.47	72.6	7.22	3.61	1.254	0.019	1.0507	1.121
GER	GER_Ta	elytra	0.0561	646.7	1083.2	3724.8	479.14	10.78	65.2	21.57	5.87	0.631	0.010	0.5960	0.288
GER	GER_Ta	elytra	0.0504	534.6	627.1	3241.2	654.17	12.22	36.9	5.04	3.32	0.626	0.008	0.4811	0.175
GER	GER_Ta	elytra	0.0521	431.0	667.5	2950.1	643.95	11.61	13.4	7.20	3.80	0.452	0.004	0.5356	0.399
GER	GER_Ta	elytra	0.0685	903.3	496.1	2210.4	1133.43	11.94	56.2	5.65	3.26	0.691	0.011	0.5254	0.473
GER	GER_Ta	elytra	0.0677	1092.2	486.0	1877.6	1012.85	11.58	77.4	5.00	2.94	0.658	0.016	0.7479	0.531
GER	GER_Ta	elytra	0.0518	364.7	540.7	2532.7	897.10	11.89	29.3	0.00	3.36	2.388	0.005	0.2739	0.430
GER	GER_Ta	elytra	0.0526	376.6	594.1	2586.9	1111.03	10.22	39.7	4.83	3.79	1.810	0.005	0.5531	1.246
GER	GER_Ta	elytra	0.0543	335.0	612.6	2688.1	913.81	8.46	27.6	5.13	3.29	1.719	0.004	0.2949	0.333
GER	GER_Ta	elytra	0.0484	360.2	562.3	2120.8	876.65	18.05	76.0	3.00	3.67	0.747	0.005	1.0770	0.910
GER	GER_Ta	elytra	0.0435	399.1	600.8	2099.1	946.67	18.54	83.2	2.50	4.09	0.766	0.007	0.4807	0.556
ITA	ITA	elytra	0.0670	640.7	869.4	978.1	456.27	25.58	37.0	6.86	2.88	1.976	0.014	0.5857	0.347
ITA	ITA	elytra	0.0674	527.4	819.3	1005.3	345.40	23.43	32.2	6.64	3.76	1.855	0.013	1.4670	0.269
ITA	ITA	elytra	0.0371	636.8	1007.2	1273.4	743.40	25.66	37.5	40.77	4.31	2.538	0.028	1.8737	0.185
ITA	ITA	elytra	0.0374	598.5	875.9	1294.8	628.07	23.66	20.3	60.18	4.71	1.357	0.027	32.5962	0.192
ITA	ITA	elytra	0.0411	625.2	653.4	2762.3	744.28	17.17	16.1	13.54	4.73	1.257	0.029	0.3165	0.271
ITA	ITA	elytra	0.0398	358.6	504.7	3141.6	632.16	18.01	13.6	9.42	4.34	1.066	0.020	0.5610	0.427
ITA	ITA	elytra	0.0424	311.8	613.0	3348.4	1204.01	18.23	19.3	44.80	4.35	1.404	0.024	0.3476	0.134
ITA	ITA	elytra	0.0416	313.3	595.4	3088.8	1041.83	19.92	18.8	25.60	4.55	1.261	0.016	0.3068	0.262
ITA	ITA	elytra	0.0715	2670.4	1644.4	977.8	340.84	16.45	193.0	12.69	3.48	0.405	0.031	0.3693	0.351
ITA	ITA	elytra	0.0691	3755.3	1689.0	981.9	279.02	31.28	289.1	19.96	4.07	1.409	0.016	0.2380	0.293
ITA	ITA	elytra	0.0494	357.4	632.6	2993.2	584.41	21.09	24.1	13.72	3.25	2.061	0.019	0.4378	0.224
ITA	ITA	abdomen	0.1473	4634.5	6416.5	5487.1	686.63	25.62	469.7	147.03	25.57	8.580	0.030	0.9919	0.536
ITA	ITA	abdomen	0.1361	560.6	5324.1	6016.0	826.16	11.44	82.8	148.66	29.63	14.716	0.019	0.1306	0.200
ITA	ITA	abdomen	0.1602	851.2	4482.7	5425.5	644.44	10.42	192.0	109.59	28.51	3.343	0.013	0.1288	0.054
ITA	ITA	abdomen	0.0851	521.8	2495.9	14,147.8	2268.98	17.64	140.4	254.30	122.20	33.032	0.029	0.1975	0.078
ITA	ITA	abdomen	0.0866	778.5	3137.7	12,337.1	1534.30	29.23	112.1	257.18	134.68	39.022	0.083	0.2300	0.421
ITA	ITA	abdomen	0.0904	1263.0	2760.5	13,792.1	2612.17	27.13	68.6	283.48	61.54	25.354	0.043	1.6034	0.082
ITA	ITA	abdomen	0.2585	837.8	5081.2	5768.2	600.85	12.35	157.0	88.33	36.92	11.197	0.013	0.0181	0.083
POL	POL_La	abdomen	0.1020	439.2	13,474.6	12,331.8	876.18	29.65	217.5	257.28	98.48	28.391	0.113	1.0509	6.041
POL	POL_La	abdomen	0.2280	266.3	5903.1	6895.8	959.39	13.26	110.5	101.80	21.72	1.573	0.020	0.2677	0.239
POL	POL_La	abdomen	0.1586	361.0	1507.2	11,696.2	2003.09	31.92	366.5	143.37	30.31	17.620	0.019	0.5575	0.298
POL	POL_La	elytra	0.0691	316.0	582.5	2376.9	1237.48	18.96	30.0	25.22	3.93	0.971	0.010	0.8483	0.933
POL	POL_La	elytra	0.0480	242.3	1074.0	2115.8	298.13	43.87	41.5	30.50	3.98	2.514	0.023	0.4189	1.383
POL	POL_La	elytra	0.0408	1765.1	725.3	4884.9	104.17	84.27	76.2	105.28	17.06	1.332	0.064	1.4069	2.000
POL	POL_La	elytra	0.0408	367.5	603.5	1570.2	407.84	33.76	50.0	12.16	3.79	0.843	0.007	0.7202	1.012
POL	POL_Łsz	abdomen	0.0643	589.0	4091.6	13,106.3	2541.52	61.31	193.2	274.19	55.88	4.350	0.067	1.2594	0.443
POL	POL_Łsz	abdomen	0.0516	419.5	1229.3	10,323.9	1718.41	65.55	177.7	280.93	41.77	13.574	0.029	1.0360	4.916
POL	POL_Łsz	abdomen	0.0899	351.2	974.7	6245.5	1068.97	23.92	98.0	195.72	62.41	12.668	0.032	0.4881	0.318
POL	POL_Łsz	abdomen	0.1149	756.9	2081.0	17,722.4	1969.71	84.12	170.4	196.57	109.65	66.853	0.044	0.6132	0.288
POL	POL_Łsz	abdomen	0.0679	1200.0	3644.5	29,765.8	4017.97	109.03	117.5	300.90	188.70	24.931	0.083	0.7238	2.342
POL	POL_Łsz	abdomen	0.1238	231.3	2157.7	9628.4	1321.00	41.71	131.7	166.47	51.01	38.805	0.024	0.4445	0.274
POL	POL_Łsz	elytra	0.0719	265.6	551.8	6520.4	1058.28	19.00	38.2	38.03	5.44	2.917	0.015	0.4172	0.346
POL	POL_Łsz	elytra	0.0733	253.2	503.9	6060.0	1016.78	20.17	38.2	34.17	5.64	2.974	0.011	0.4108	0.216
POL	POL_Łsz	elytra	0.0652	315.8	476.0	3497.0	706.75	13.57	17.5	10.76	3.71	0.640	0.011	0.4569	0.612
POL	POL_Łsz	elytra	0.0656	303.2	447.8	3665.6	654.73	14.17	19.1	13.10	3.11	0.772	0.008	0.3479	0.305
POL	POL_Łsz	elytra	0.0650	255.3	602.6	3309.6	540.00	20.16	18.6	15.45	6.36	1.364	0.008	0.3059	0.252
POL	POL_Łsz	elytra	0.0643	260.5	574.4	3459.3	557.54	17.42	18.2	18.44	4.34	1.597	0.010	0.4306	0.375
POL	POL_Łsz	elytra	0.0629	266.7	452.1	5967.4	1084.74	16.03	41.2	44.05	7.50	7.106	0.018	0.7016	0.798
POL	POL_Łsz	elytra	0.0608	251.6	464.2	6522.9	1158.06	16.03	45.1	43.98	7.41	7.019	0.011	0.4895	0.503
POL	POL_Łsz	elytra	0.0582	334.2	587.6	3413.5	667.87	8.27	16.0	10.60	4.74	2.257	0.008	0.5473	1.020
POL	POL_Łsz	elytra	0.0585	313.1	594.5	3260.4	745.64	6.89	17.8	7.65	3.76	1.946	0.007	0.5552	0.387
POL	POL_Łsz	elytra	0.0691	321.6	576.2	4267.4	664.69	14.91	31.1	8.23	3.95	0.771	0.005	0.2691	0.747
POL	POL_Łsz	elytra	0.0716	383.3	641.6	4464.1	742.88	16.89	39.1	11.83	4.81	0.794	0.003	0.2426	0.065
POL	POL_Mś	elytra	0.0880	246.7	490.9	4107.7	725.68	26.98	44.9	17.05	4.26	2.120	0.021	0.6794	0.430
POL	POL_Mś	elytra	0.0880	848.1	625.1	4969.1	975.00	26.35	23.6	22.28	5.61	4.180	0.028	4.7553	0.264
POL	POL_Mś	abdomen	0.1350	469.4	1379.7	12,022.9	2022.89	28.12	130.4	212.46	123.64	79.567	0.045	0.6985	0.203
POL	POL_Mś	elytra	0.0710	705.3	454.3	4088.0	944.37	12.46	31.4	32.38	4.80	0.252	0.098	0.7073	0.336
POL	POL_Mś	elytra	0.0710	254.0	421.9	3252.4	771.83	14.20	33.9	33.57	4.71	0.293	0.058	30.7702	0.507
POL	POL_Mś	abdomen	0.1400	456.4	1970.2	17,210.4	1954.57	52.96	102.5	211.89	155.35	4.608	0.052	0.6218	0.152
POL	POL_Mś	elytra	0.0760	283.6	524.8	3355.2	1088.29	21.22	36.2	17.04	4.53	0.745	0.023	0.9354	0.318
POL	POL_Mś	elytra	0.0690	289.7	470.6	3297.3	1073.62	18.67	280.3	21.22	4.52	1.524	0.044	0.7985	0.640
POL	POL_Mś	abdomen	0.1200	662.5	1913.4	11,369.6	1899.75	35.75	203.3	232.83	181.24	22.776	0.021	0.5928	0.219
POL	POL_Prz	elytra	0.0640	2444.6	599.6	4218.1	1148.44	25.03	48.8	15.50	129.60	0.556	0.025	0.5299	0.195
POL	POL_Prz	elytra	0.0640	2177.7	598.5	3614.2	1089.06	21.35	22.8	31.38	5.15	0.848	0.030	0.6272	0.153
POL	POL_Prz	abdomen	0.1380	434.0	3247.3	16,084.5	1783.12	45.53	81.6	167.24	73.48	28.065	0.050	0.6886	0.107
POL	POL_Prz	elytra	0.0570	334.3	606.3	3932.8	1269.47	22.60	22.1	13.80	5.37	0.878	0.048	0.7095	0.410
POL	POL_Prz	elytra	0.0577	297.3	462.9	3809.4	1233.80	24.65	34.0	23.91	5.13	0.459	0.118	1.3035	0.181
POL	POL_Prz	abdomen	0.0917	512.5	2551.8	18,606.7	2081.57	49.95	108.4	232.80	85.48	22.753	0.052	0.8150	0.074
POL	POL_Prz	elytra	0.0734	292.9	594.4	2321.9	497.41	20.90	17.2	18.63	3.46	0.439	0.048	1.7877	0.036
POL	POL_Prz	elytra	0.0766	286.9	518.9	1975.3	434.60	20.47	13.6	6.32	4.67	0.382	0.020	0.3259	0.020
POL	POL_Prz	abdomen	0.0658	216.4	3194.0	1692.6	144.53	137.53	170.5	54.62	22.89	0.000	0.043	2.4831	0.234
POL	POL_Prz	elytra	0.0641	324.9	764.9	3420.4	1414.98	28.48	16.5	14.91	5.33	1.541	0.012	0.2381	0.127
POL	POL_Prz	elytra	0.0628	361.6	671.8	5485.5	1725.16	19.62	15.4	14.26	6.41	12.694	0.021	0.2597	0.071
POL	POL_Prz	elytra	0.0339	468.1	598.9	3048.7	743.36	23.13	13.3	32.12	4.50	1.375	0.029	0.6217	0.291
POL	POL_Prz	elytra	0.0336	338.6	600.0	3229.5	742.56	24.33	12.8	19.09	4.54	1.538	0.020	0.4132	0.262
POL	POL_Prz	elytra	0.0544	367.4	571.8	4941.4	1513.42	16.06	23.0	13.35	4.49	1.247	0.015	0.2983	0.177
POL	POL_Prz	elytra	0.0554	275.2	423.7	4391.4	1031.05	19.21	12.3	7.64	3.54	0.848	0.012	0.3318	0.115
POL	POL_Prz	elytra	0.0638	287.2	764.0	3941.2	1347.18	21.94	15.7	9.86	3.37	1.225	0.014	0.2456	0.077
POL	POL_Prz	elytra	0.0659	336.5	565.9	4911.4	1496.97	19.54	14.7	9.92	5.86	7.981	0.015	0.3428	0.123
POL	POL_Prz	elytra	0.0601	241.8	620.6	3362.7	707.49	17.89	13.6	17.92	5.14	4.001	0.015	0.2656	0.078
POL	POL_Prz	elytra	0.0586	219.3	421.4	3336.7	600.68	17.58	14.3	15.49	3.61	4.294	0.020	0.2793	0.086
POL	POL_Prz	elytra	0.0684	278.4	371.6	2983.5	1037.57	25.22	11.1	7.08	4.27	1.496	0.008	0.1517	0.023
POL	POL_Prz	elytra	0.0674	314.9	336.5	3116.9	1012.61	24.59	8.9	5.39	3.48	1.587	0.008	0.1424	0.063
POL	POL_Prz	elytra	0.0460	381.9	491.5	3116.9	851.74	23.37	13.9	11.57	4.52	2.397	0.036	0.3795	0.213
POL	POL_Prz	elytra	0.0496	498.1	512.4	3263.1	919.96	21.45	28.0	21.96	4.86	2.784	0.023	0.5546	0.224
POL	POL_Prz	elytra	0.0590	285.4	410.0	3518.2	793.22	20.12	20.5	20.30	4.87	1.163	0.017	0.3138	0.137
POL	POL_Prz	elytra	0.0597	443.5	413.7	3385.9	783.58	23.64	15.9	16.82	4.16	1.096	0.018	0.2139	0.140
POL	POL_Prz	elytra	0.0464	351.1	518.3	2459.4	654.09	11.10	11.2	13.56	4.28	0.771	0.049	0.2240	0.370
POL	POL_Prz	elytra	0.0462	296.7	462.9	2429.8	666.45	13.58	13.4	49.24	6.77	0.608	0.040	0.9260	0.052
POL	POL_Prz	elytra	0.0374	380.5	529.4	3143.3	750.00	11.98	23.5	16.50	3.63	1.484	0.018	0.3127	0.275
POL	POL_Prz	elytra	0.0367	1281.8	549.3	3235.1	781.20	11.90	12.5	36.27	4.76	1.233	0.026	1.9037	0.183
POL	POL_Prz	abdomen	0.1318	568.7	4245.2	14,296.6	2677.85	78.69	78.6	214.99	98.51	50.441	0.034	0.4306	0.169
POL	POL_Prz	abdomen	0.0822	754.2	5599.7	20,070.5	2137.35	27.80	54.0	320.87	54.58	28.896	0.035	0.5190	0.062
POL	POL_Prz	abdomen	0.1246	635.0	2060.0	14,449.5	2218.46	96.72	51.4	222.01	75.74	40.225	0.077	2.5747	0.225
POL	POL_Prz	abdomen	0.1465	340.8	1849.9	16,010.3	1960.20	33.56	85.4	194.95	78.28	67.542	0.025	0.3153	0.037
POL	POL_Prz	abdomen	0.0950	445.6	2361.6	15,762.7	2139.58	122.49	82.7	263.85	69.39	71.503	0.050	0.6480	0.073
POL	POL_Prz	abdomen	0.1010	503.4	4772.7	16,602.0	2092.48	187.07	193.0	250.73	58.76	66.145	0.047	0.5702	0.102
POL	POL_Prz	abdomen	0.0934	431.8	2077.5	13,743.8	1563.60	126.27	119.7	219.62	32.10	48.667	0.046	0.7247	1.154
POL	POL_Prz	abdomen	0.1217	434.0	1451.2	9602.1	1940.84	27.88	93.1	198.29	84.42	78.045	0.033	3.4367	0.164
POL	POL_Prz	abdomen	0.1020	513.5	2653.4	19,667.5	2275.78	71.70	70.8	251.02	88.80	27.575	0.046	0.2402	0.031
POL	POL_Prz	abdomen	0.0786	592.2	3123.7	20,761.3	2375.95	45.60	45.8	319.37	26.89	42.454	0.031	0.4588	0.086
POL	POL_Prz	abdomen	0.0271	1279.3	956.5	1126.3	120.30	623.23	431.0	303.62	59.15	2.911	0.184	13.0152	1.736
POL	POL_Prz	abdomen	0.0409	1300.3	1638.9	1168.7	81.17	501.95	314.7	457.76	84.43	7.211	0.135	9.9166	1.486
POL	POL_Prz	elytra	0.0873	273.4	396.7	2745.2	641.47	37.85	39.6	28.97	4.56	1.392	0.011	0.4356	0.227
POL	POL_Prz	elytra	0.0914	288.0	593.2	2829.8	1110.39	36.27	23.9	18.80	3.72	1.319	0.010	0.3638	1.252
POL	POL_Prz	elytra	0.0514	487.1	149.9	558.9	82.10	320.96	134.0	54.14	9.14	0.439	0.086	4.2312	0.684
POL	POL_Prz	elytra	0.0515	422.9	120.2	529.3	96.31	270.32	61.4	36.65	7.29	0.535	0.082	2.3340	1.811
POL	POL_PrzB	elytra	0.0530	337.6	1152.0	8913.1	593.58	35.29	310.0	45.20	19.35	0.000	0.000	2.5428	1.018
POL	POL_PrzB	elytra	0.0540	572.8	1124.0	2793.8	660.00	30.49	75.7	31.82	10.88	2.967	0.212	2.3462	1.118
POL	POL_PrzB	abdomen	0.0830	297.3	3037.9	8778.8	1071.33	34.95	382.3	212.24	104.43	36.046	0.150	2.4167	0.677
POL	POL_PrzB	elytra	0.0300	428.1	672.0	3053.7	564.33	28.37	226.7	19.36	6.59	1.458	0.108	2.1102	1.237
POL	POL_PrzB	elytra	0.0300	539.1	559.2	3261.3	694.33	29.49	169.0	21.78	6.11	1.487	0.048	3.1622	1.445
POL	POL_PrzB	abdomen	0.0740	476.9	1592.8	14,846.0	1544.73	44.19	412.7	306.14	61.33	29.163	0.115	2.0573	1.677
POL	POL_Wł	abdomen	0.1276	493.4	2311.2	15,077.2	2135.89	26.42	216.0	208.86	132.64	17.939	0.023	1.1149	0.223
POL	POL_Wł	abdomen	0.1155	604.5	4769.5	9841.4	2410.48	53.04	120.8	226.85	69.49	27.415	0.020	0.6603	0.163
POL	POL_Wł	abdomen	0.2116	755.4	9191.9	10,237.3	1633.27	39.70	111.1	123.28	49.67	13.757	0.003	0.4840	0.015
POL	POL_Wł	abdomen	0.0899	327.5	2633.3	14,898.4	1569.74	27.16	128.7	272.97	211.23	144.664	0.019	0.9300	0.188
POL	POL_Wł	abdomen	0.0392	356.8	780.6	3625.7	791.33	56.86	105.4	72.85	31.94	11.605	0.019	1.3542	3.047
POL	POL_Wł	abdomen	0.0446	485.3	2192.3	5002.4	1008.30	48.72	128.0	228.16	38.90	20.581	0.051	0.9931	0.768
POL	POL_Wł	elytra	0.0597	382.8	723.6	4511.0	1744.72	31.52	33.8	6.49	6.01	2.575	0.014	0.7549	0.533
POL	POL_Wł	elytra	0.0571	389.2	645.6	4010.6	1418.74	33.15	21.7	4.87	4.89	2.483	0.009	0.6777	0.465
POL	POL_Wł	elytra	0.0758	271.9	451.2	3829.9	1266.89	19.36	19.9	6.39	3.97	1.113	0.006	0.3241	0.312
POL	POL_Wł	elytra	0.0758	309.4	552.8	3920.4	1267.68	19.80	21.5	10.06	4.31	1.189	0.008	0.4438	0.264
POL	POL_Wł	elytra	0.0819	229.5	533.6	2820.3	1360.93	20.38	20.9	5.17	3.99	0.766	0.006	0.3633	0.737
POL	POL_Wł	elytra	0.0849	239.8	491.9	2721.6	1368.08	19.79	18.3	7.70	3.79	0.812	0.014	0.4090	0.227
POL	POL_Wł	elytra	0.0686	413.0	741.0	3061.8	1056.12	28.57	30.9	6.53	7.59	1.249	0.005	0.3796	0.206
POL	POL_Wł	elytra	0.0701	381.4	669.7	2532.3	953.50	30.04	32.7	5.18	6.52	1.223	0.010	0.4297	0.241
POL	POL_Wł	elytra	0.0510	410.5	872.5	4616.5	1363.73	34.48	37.1	9.49	3.64	1.186	0.011	0.5091	0.285
POL	POL_Wł	elytra	0.0456	513.2	530.5	2511.6	701.75	41.51	50.9	10.35	6.27	1.790	0.047	0.6265	0.582
RUS	RUS	abdomen	0.1410	922.7	1534.5	9521.6	2356.38	19.14	124.2	180.81	52.42	2.482	0.023	0.3100	0.217
RUS	RUS	elytra	0.0779	318.6	583.2	2965.1	2212.45	9.63	19.1	12.12	4.29	0.189	0.004	0.3014	0.221
RUS	RUS	elytra	0.0788	262.0	505.3	2650.6	1571.45	7.25	28.9	11.98	4.20	0.193	0.006	0.2247	0.158
SLO	Slo_GB	abdomen	0.1071	567.9	391.3	1018.2	196.83	14.75	113.0	27.57	47.75	1.295	0.016	0.1729	0.737
SLO	Slo_GB	elytra	0.0776	355.8	163.3	846.2	130.54	11.11	25.6	9.04	8.66	0.417	0.008	0.1936	0.366
SLO	Slo_GB	elytra	0.0774	289.3	163.7	649.3	119.25	9.98	20.8	7.82	6.03	0.435	0.005	0.1523	1.146
SLO	Slo_GB	abdomen	0.0804	437.1	651.9	789.5	257.96	14.35	152.7	103.24	37.85	23.144	0.010	0.3318	0.384
SLO	Slo_GB	elytra	0.0532	166.6	154.3	420.3	121.62	7.79	25.4	29.11	5.27	6.078	0.003	0.3255	0.302
SLO	Slo_GB	elytra	0.0546	212.0	143.7	404.5	137.00	8.21	26.2	22.38	6.57	5.140	0.007	0.3360	0.326
SLO	Slo_MS1	abdomen	0.1065	290.0	414.4	688.0	188.36	20.40	817.7	55.67	40.87	12.564	0.039	0.5504	0.297
SLO	Slo_MS1	elytra	0.0802	255.7	269.3	688.9	183.29	11.31	42.5	30.17	5.10	4.919	0.009	0.2023	0.799
SLO	Slo_MS1	elytra	0.0780	207.7	190.2	464.0	119.87	11.49	36.3	25.75	5.30	4.429	0.009	0.2186	0.526
SLO	Slo_MS3	abdomen	0.0828	623.7	662.6	2201.5	409.90	18.94	126.7	56.99	53.52	6.032	0.078	0.2698	0.349
SLO	Slo_MS3	elytra	0.0556	434.1	310.8	1245.3	240.29	13.29	34.7	36.13	5.78	2.593	0.023	0.2741	0.515
SLO	Slo_MS3	elytra	0.0544	284.6	251.5	1164.3	216.73	12.56	25.2	34.48	5.42	2.421	0.013	0.1871	0.245
SLO	Slo_MS3	abdomen	0.1012	621.9	823.2	2745.8	445.95	27.00	286.4	71.50	39.71	1.992	0.057	0.2154	1.126
SLO	Slo_MS3	elytra	0.0689	372.1	366.8	1608.5	351.52	21.94	23.9	34.07	7.88	1.139	0.019	0.2426	4.396
SLO	Slo_MS3	elytra	0.0702	330.8	331.3	1331.3	226.64	21.54	23.8	27.23	7.10	1.021	0.013	0.2545	0.484
SLO	Slo_MS3	abdomen	0.0928	554.5	577.2	2341.1	439.87	15.09	170.4	71.06	32.32	18.900	0.080	0.3108	0.421
SLO	Slo_MS3	elytra	0.0494	248.1	161.8	898.2	177.33	11.34	32.6	20.57	7.45	5.770	0.015	0.4200	0.434
SLO	Slo_MS3	elytra	0.0529	295.3	156.5	922.3	183.55	11.01	35.5	17.16	8.03	4.837	0.020	1.7953	1.053
SLO	Slo_MS3	abdomen	0.0676	579.3	503.8	2880.4	452.22	15.91	120.4	45.64	59.13	5.855	0.105	0.3433	0.158
SLO	Slo_MS3	elytra	0.0455	331.3	185.1	1118.6	197.36	9.60	26.6	10.37	5.63	1.614	0.015	0.5365	0.169
SLO	Slo_MS3	elytra	0.0480	336.6	229.5	1329.0	230.00	10.97	38.5	18.15	12.03	2.090	0.017	0.7822	0.579
SLO	Slo_MS3	abdomen	0.0786	519.5	424.1	2257.0	358.27	21.94	130.4	43.87	43.21	5.107	0.088	0.3276	0.553
SLO	Slo_MS3	elytra	0.0656	253.3	185.5	1036.4	206.40	17.93	33.4	17.89	5.70	1.367	0.014	0.3528	0.472
SLO	Slo_MS3	elytra	0.0656	255.5	187.7	938.3	165.40	20.83	47.1	20.11	6.40	1.465	0.011	0.3969	0.906
SPA	SPA_Ho	abdomen	0.0805	534.9	1871.1	8327.1	1543.85	33.53	935.5	284.98	75.17	0.974	0.020	0.6115	0.711
SPA	SPA_Ho	abdomen	0.0439	477.9	3957.5	5153.2	1010.02	25.77	182.7	159.31	46.27	69.977	0.051	0.7557	0.269
SPA	SPA_Ho	abdomen	0.1032	606.2	1813.3	12,765.2	1241.86	23.01	131.8	172.59	73.85	29.170	0.059	0.1918	0.401
SPA	SPA_Ho	abdomen	0.0536	384.7	4954.0	9203.6	1247.95	42.63	759.3	314.99	59.64	2.654	0.093	0.7476	0.617
SPA	SPA_Ho	elytra	0.0510	412.6	371.3	2055.3	371.57	22.40	267.5	25.62	4.05	0.348	0.017	0.6301	0.553
SPA	SPA_Ho	elytra	0.0282	642.4	926.8	2250.6	923.40	17.08	152.1	30.46	6.12	1.530	0.034	0.8010	0.619
SPA	SPA_Ho	elytra	0.0283	276.7	1007.5	4658.9	1040.99	23.35	175.3	29.50	5.03	1.905	0.017	0.7359	0.326
SPA	SPA_Ho	elytra	0.0413	283.9	530.0	3389.1	937.77	13.83	145.3	17.58	5.51	2.652	0.005	0.6743	0.846
SPA	SPA_Ho	elytra	0.0413	354.8	599.7	3163.4	969.25	14.92	90.8	19.04	4.68	3.101	0.008	0.4848	0.169
SPA	SPA_Ho	elytra	0.0473	296.6	978.8	1993.2	997.67	16.81	113.5	16.12	4.51	4.800	0.015	0.4441	0.172
SPA	SPA_Ho	elytra	0.0459	267.1	992.9	2077.2	1044.66	17.57	123.5	19.51	6.95	37.268	0.070	0.6755	0.370
SPA	SPA_La	abdomen	0.0825	534.5	3069.4	5413.5	834.67	21.72	158.3	117.04	19.44	2.351	0.586	0.3570	0.192
SPA	SPA_La	abdomen	0.0693	686.5	3114.8	5664.1	1238.67	21.33	380.4	139.68	46.02	32.164	0.087	0.6168	0.112
SPA	SPA_La	abdomen	0.0771	513.6	2924.8	8711.0	2666.15	22.52	173.8	345.39	80.06	16.812	0.123	1.2133	0.107
SPA	SPA_La	abdomen	0.0619	368.7	1755.2	4691.8	878.51	19.36	231.0	86.60	28.75	31.481	0.136	0.8473	0.132
SPA	SPA_La	abdomen	0.1038	483.5	5246.7	6035.4	1288.54	18.88	95.8	144.98	21.05	0.519	0.059	0.5672	1.111
SPA	SPA_La	abdomen	0.1100	402.9	1898.8	7263.1	1397.09	8.96	142.3	214.83	33.56	83.527	0.078	0.4659	0.194
SPA	SPA_La	abdomen	0.0931	384.0	7268.8	5922.6	983.57	10.95	42.7	189.39	8.63	1.231	0.094	0.6644	0.277
SPA	SPA_La	abdomen	0.0725	407.4	3247.4	5081.8	933.24	16.84	819.7	126.17	37.95	7.964	0.067	1.1002	0.381
SPA	SPA_La	elytra	0.0572	273.9	697.3	2175.0	1135.31	11.94	39.9	31.52	3.82	0.822	0.050	0.6677	0.183
SPA	SPA_La	elytra	0.0592	318.5	656.8	2516.8	1168.75	11.16	52.5	35.36	2.96	0.771	0.029	9.8121	0.188
SPA	SPA_La	elytra	0.0567	442.5	758.1	1370.8	717.81	32.99	34.6	156.64	2.88	0.087	0.202	0.3599	0.081
SPA	SPA_La	elytra	0.0546	382.7	710.8	1539.3	715.20	22.77	24.7	7.31	3.93	0.042	0.029	0.5387	0.318
SPA	SPA_La	elytra	0.0326	469.9	777.4	2130.2	820.55	15.46	50.0	7.79	7.00	1.120	0.055	2.4871	0.209
SPA	SPA_La	elytra	0.0358	453.8	915.1	1334.9	505.87	22.21	182.1	107.14	9.06	1.760	0.120	2.2592	0.133
SPA	SPA_La	elytra	0.0467	451.9	985.2	1359.6	718.63	13.67	29.1	27.21	4.50	0.208	0.056	1.1245	1.583
SPA	SPA_La	elytra	0.0498	415.3	964.3	1559.0	719.08	13.94	33.3	10.69	5.10	0.191	0.041	0.7343	0.059
SPA	SPA_La	elytra	0.0413	329.3	707.8	2737.9	1212.35	7.86	32.9	10.25	5.83	4.404	0.040	0.8421	0.484
SPA	SPA_La	elytra	0.0408	367.7	661.8	2553.5	1239.46	9.33	27.0	9.79	4.65	4.627	0.034	0.8224	0.081
SPA	SPA_La	elytra	0.0308	509.9	1016.9	1147.2	486.36	16.36	68.8	3.54	4.40	0.249	0.035	0.9699	0.020
SPA	SPA_La	elytra	0.0353	430.9	979.0	2098.4	810.20	10.47	55.8	13.71	8.37	1.657	0.021	0.5915	1.549
SPA	SPA_La	elytra	0.0641	316.8	795.3	1668.3	356.63	19.92	41.3	14.16	3.90	0.380	0.024	0.4708	0.102
SPA	SPA_La	elytra	0.0415	384.3	959.4	1827.8	419.28	22.67	99.8	7.29	5.57	1.616	0.028	0.8376	0.165
SPA	SPA_La	elytra	0.0355	534.4	1040.5	1381.3	520.56	26.19	322.5	19.09	5.88	2.379	0.082	3.0525	0.259
SPA	SPA_La	elytra	0.0480	271.5	810.0	1198.2	435.83	14.93	175.4	16.89	4.90	5.108	0.055	0.8011	0.161
SPA	SPA_La	elytra	0.0286	463.8	971.7	854.5	391.61	16.84	82.2	4.23	4.73	0.256	0.044	1.1800	1.154
SPA	SPA_LZ	abdomen	0.0413	399.0	1258.7	5145.2	1084.26	42.03	372.2	156.45	54.43	254.859	0.061	0.7238	0.371
SPA	SPA_LZ	abdomen	0.1067	556.4	5496.8	9316.9	1065.14	21.31	161.1	123.97	48.80	119.494	0.042	0.3462	0.143
SPA	SPA_LZ	abdomen	0.0941	391.3	6007.1	9947.4	1707.86	32.02	223.9	195.66	76.14	26.769	0.016	0.3153	0.157
SPA	SPA_LZ	elytra	0.0558	335.3	609.0	2511.0	1660.93	23.28	78.9	23.85	5.19	20.515	0.014	0.3123	0.066
SPA	SPA_LZ	elytra	0.0361	252.5	1114.9	2745.5	794.74	18.61	88.9	15.75	4.62	7.919	0.051	0.6185	0.466
SPA	SPA_LZ	elytra	0.0362	222.3	1091.9	2647.4	678.45	20.11	92.5	16.38	4.85	7.831	0.065	0.3825	0.679
SPA	SPA_LZ	elytra	0.0464	359.0	1177.8	1853.3	706.90	21.72	108.0	26.60	4.71	1.450	0.025	0.4267	0.189
SPA	SPA_LZ	elytra	0.0430	278.2	1031.4	1634.7	731.16	20.58	99.1	29.27	4.41	1.477	0.025	0.6081	5.960
UK	UK_Clo	abdomen	0.0466	1100.5	1792.3	2883.2	574.46	33.65	161.8	286.41	63.38	1.876	0.129	3.9180	0.861
UK	UK_Clo	elytra	0.0493	395.1	639.7	2885.8	1410.75	17.95	73.2	41.72	6.58	0.981	0.089	2.3256	0.613
UK	UK_Clo	elytra	0.0490	335.9	642.1	2891.8	1328.98	18.06	53.1	41.98	8.99	0.762	0.093	2.0166	0.477
UK	UK_Clo	abdomen	0.0486	781.5	4165.9	14,496.1	2736.42	20.28	827.2	422.01	89.04	231.982	0.079	32.6688	0.220
UK	UK_Clo	elytra	0.0516	343.3	740.9	1985.0	1131.78	17.58	26.2	11.49	4.24	0.561	0.075	0.6464	0.003
UK	UK_Clo	elytra	0.0540	302.8	773.3	2274.8	1204.26	14.52	32.2	12.77	4.36	0.782	0.020	0.3733	0.052
UK	UK_Clo	abdomen	0.0350	1315.0	5519.3	7025.6	986.57	53.12	1666.6	495.40	89.83	8.863	0.069	17.8577	1.100
UK	UK_Clo	abdomen	0.0480	1897.1	16,348.5	8371.2	702.08	70.93	2522.3	483.37	105.85	9.517	0.129	20.5830	2.242
UK	UK_Clo	abdomen	0.0272	999.6	3703.2	8524.1	1559.93	44.06	351.5	193.07	48.65	159.601	0.048	6.2771	0.424
UK	UK_Clo	abdomen	0.0275	2004.5	4911.7	5612.2	902.18	48.47	199.3	658.58	73.97	56.606	0.107	4.2580	2.040
UK	UK_Clo	abdomen	0.0159	2684.7	720.0	2231.3	555.97	26.77	280.5	116.43	70.56	57.387	0.161	10.7706	1.252
UK	UK_Clo	elytra	0.1680	70.8	70.3	207.2	50.89	1.93	7.8	0.65	1.33	1.317	0.007	0.2569	0.051
UK	UK_Clo	elytra	0.1690	155.2	24.3	208.5	37.46	2.58	12.1	3.58	1.23	0.865	0.021	0.3143	0.050
UK	UK_Clo	elytra	0.0256	460.2	677.8	5760.6	886.72	17.06	75.0	12.76	4.89	18.799	0.053	1.3076	0.049
UK	UK_Clo	elytra	0.0250	378.0	676.8	4586.4	919.20	16.58	160.4	22.26	11.58	25.553	0.053	2.3187	0.010
UK	UK_Clo	elytra	0.0313	322.3	825.8	5203.7	1146.33	7.51	139.6	61.85	9.54	45.001	0.040	3.6947	0.088
UK	UK_Clo	elytra	0.0344	711.4	1023.5	3569.0	600.00	18.88	141.6	58.39	13.75	2.467	0.041	4.9854	0.584
UK	UK_Clo	elytra	0.0412	238.8	1080.0	1781.5	881.31	9.51	35.0	7.34	4.18	3.032	0.041	0.8180	0.578
UK	UK_Clo	elytra	0.0407	248.6	889.8	1626.8	673.96	9.36	24.3	24.38	3.99	3.181	0.021	1.2505	0.568
UK	UK_Clo	elytra	0.0260	542.8	689.5	2798.0	480.77	22.83	50.0	75.39	15.03	8.993	0.049	1.1148	0.627
UK	UK_Clo	elytra	0.3120	27.6	60.0	382.0	95.99	0.75	10.5	3.68	7.73	2.857	0.004	0.2265	0.023
UK	UK_Fet	abdomen	0.0538	1680.7	3248.0	20,611.8	5717.10	51.21	648.1	390.42	167.02	80.656	0.058	1.7777	0.479
UK	UK_Fet	abdomen	0.0718	1043.8	2869.0	15,644.2	2090.95	26.99	188.2	340.33	146.18	52.155	0.111	0.6883	0.229
UK	UK_Fet	abdomen	0.0536	1250.3	3957.3	13,328.4	2685.45	43.67	449.1	480.14	101.64	67.574	0.042	3.7169	0.492
UK	UK_Fet	abdomen	0.0685	977.4	3220.6	17,242.0	2867.59	30.08	266.6	387.99	251.70	134.733	0.061	1.8056	0.428
UK	UK_Fet	abdomen	0.0790	891.3	5602.3	11,191.2	3061.27	22.97	268.4	330.40	77.44	75.361	0.038	2.0241	0.252
UK	UK_Fet	abdomen	0.0581	1160.6	4374.5	15,183.8	3221.17	24.48	300.2	371.35	74.91	35.543	0.089	1.6312	4.489
UK	UK_Fet	abdomen	0.0495	1219.5	8756.4	11,694.2	2335.76	37.79	214.1	493.05	81.18	93.847	0.026	2.3648	0.873
UK	UK_Fet	elytra	0.0548	373.6	576.8	7172.3	2027.74	16.55	69.3	60.94	9.55	12.386	0.014	0.5418	0.468
UK	UK_Fet	elytra	0.0550	322.0	501.4	6215.2	1736.73	16.90	67.1	49.72	8.61	10.614	0.014	0.4104	1.901
UK	UK_Fet	elytra	0.0306	339.1	498.8	2384.6	937.58	21.23	30.1	37.57	7.02	1.863	0.016	0.6892	0.526
UK	UK_Fet	elytra	0.0302	299.2	503.0	2300.4	972.19	19.28	42.4	46.08	19.46	1.943	0.016	0.7337	0.422
UK	UK_Fet	elytra	0.0372	410.6	571.0	4141.1	1037.10	9.03	53.2	13.66	5.70	2.646	0.013	0.7170	0.569
UK	UK_Fet	elytra	0.0364	258.9	488.6	3640.4	937.64	9.85	36.8	14.29	11.84	2.339	0.013	0.5611	0.423
UK	UK_Fet	elytra	0.0409	325.0	447.1	3870.4	611.49	10.95	43.5	31.36	8.82	2.812	0.031	0.9526	0.679
UK	UK_Fet	elytra	0.0402	371.4	456.7	4109.9	704.98	9.75	40.3	26.19	4.53	3.340	0.023	0.4697	4.962
UK	UK_Fet	elytra	0.0407	261.2	435.2	4766.6	1022.60	11.56	56.5	42.81	4.71	5.217	0.009	0.7917	0.715
UK	UK_Fet	elytra	0.0308	275.3	556.4	3031.8	940.26	12.00	29.5	36.14	9.49	3.757	0.014	0.5724	0.688
UK	UK_Fet	elytra	0.0438	659.0	549.0	3571.1	987.44	20.71	99.3	18.23	7.33	6.316	0.016	1.9871	2.677
UK	UK_Fet	elytra	0.0431	333.7	487.8	3442.7	1008.35	14.55	48.5	31.72	5.40	4.793	0.019	0.6385	0.595

## Appendix B Supplementary materials

Image, application 1
